# KLF15 suppresses tumor growth and metastasis in Triple-Negative Breast Cancer by downregulating CCL2 and CCL7

**DOI:** 10.1038/s41598-022-23750-4

**Published:** 2022-11-08

**Authors:** Quist Kanyomse, Xin Le, Jun Tang, Fengsheng Dai, Youchaou Mobet, Chang Chen, Zhaobo Cheng, Chaoqun Deng, Yijiao Ning, Renjie Yu, Xiaohua Zeng, Tingxiu Xiang

**Affiliations:** 1 Department of Oncology, The First Affiliated Hospital of Chongqing Medical University, Chongqing, 400016 China; 2 Chongqing Key Laboratory of Molecular Oncology and Epigenetics, The First Affiliated Hospital of Chongqing Medical University, Chongqing, 400016 China; 3 Institute of Life Sciences, Chongqing Medical University, Chongqing, 400016 China; 4 Chongqing Key Laboratory of Translational Research for Cancer Metastasis and Individualized Treatment, Chongqing University Cancer Hospital, Chongqing, 400030 China

**Keywords:** Cancer, Cell biology, Molecular biology, Biomarkers

## Abstract

Kruppel like factor 15 (KLF15), a transcriptional factor belonging to the Kruppel-like factor (KLF) family of genes, has recently been reported as a tumor suppressor gene in breast cancer. However, the specific mechanisms by which KLF15 inhibits BrCa have not been elucidated. Here we investigated the role and mechanism of KLF15 in triple-negative breast cancer (TNBC). KLF15 expression and methylation were detected by RT-qPCR, RT-PCR and methylation-specific PCR in breast cancer cell lines and tissues. The effects of KLF15 on TNBC cell functions were examined via various cellular function assays. The specific anti-tumor mechanisms of KLF15 were further investigated by RNA sequence, RT-qPCR, Western blotting, luciferase assay, ChIP, and bioinformatics analysis. As the results showed that KLF15 is significantly downregulated in breast cancer cell lines and tissues, which promoter methylation of KLF15 partially contributes to. Exogenous expression of KLF15 induced apoptosis and G2/M phase cell cycle arrest, suppressed cell proliferation, metastasis and in vivo tumorigenesis of TNBC cells. Mechanism studies revealed that KLF15 targeted and downregulated C–C motif chemokine ligand 2 (CCL2) and CCL7. Moreover, transcriptome and metabolome analysis revealed that KLF15 is involved in key anti-tumor regulatory and metabolic pathways in TNBC. In conclusion, KLF15 suppresses cell growth and metastasis in TNBC by downregulating CCL2 and CCL7. KLF15 may be a prognostic biomarker in TNBC.

## Introduction

According to GLOBOCAN report published in 2021, breast cancer (BrCa) became the most common cancer globally, accounting for 1.7% of all new annual cancer cases worldwide^1^ and 15% of all cancer deaths among females^2^. Triple-negative breast cancer (TNBC) is a breast cancer subtype described as hormone receptor-negative (estrogen receptor and progesterone receptor negative) and HER2 negative^3^. This pathotype is more common in women bearing BRCA mutations (especially BRCA1)^4–7^. About 10–20% breast tumors are triple-negative. TNBC is usually characterized by poor prognosis and high recurrence rate^8^. Hence, clinical challenges of breast cancer patients treatment remain and it is vital to identify new biomarkers for comprehensive therapy. The Krüppel-like factor (KLF) family consists of transcription factors that can activate or repress different genes regulating biofunctions such as adipogenesis, heart development, erythropoiesis, fetal differentiation, and are implicated in a host of disease development including cancer^9^. KLF15, a member of KLF family has been reported involved in various diseases including cardiac hypertrophy^10^, diabetes^11^, muscle atrophy^12^ and some cancer types^13–15^. Currently, limited amount of work has been conducted discussing the activity and mechanism KLF15 in BrCa tumorigenicity. Report by Tomomi et al. suggested that KLF15 functions as a potential tumor suppressor in BrCa^15^, however its antitumor mechanism is yet to be expatiated.

Chemokines are a group of small secreted cytokines capable of regulating tumor growth and progression. Varied chemokine expression levels dictate leukocyte recruitment and activation, angiogenesis, cancer cell proliferation, and metastasis in all stages of malignancies^16,17^. In breast cancer, CCL2 and CCL7 have been reported to promote tumor malignancy^18,19^. Also, high levels of CCL2 and CCL7 in breast tumor result in poor prognosis among patients. Several reports have established a strong correlation between high expression of CCL2 and CCL7 and low survival rate amongst patients^20,21^.

TNBCs were characterized as the most malignancy among all the subtypes, therefore we determined to concentrate on this particular phenotype. In this study, we unveiled that KLF15 suppresses TNBC tumorigenicity by means of suppressing cellular proliferation, promoting cellular apoptosis and cell cycle arrest, and inhibiting migration and invasion. Xenograft tumor model established with nude mice confirmed the anti-proliferation ability of KLF15 in vivo. Moreover, Chemokines CCL2 and CCL7 were screened out as critical downstream regulators in KLF15 involved anti-cancerous effects on TNBC cells. In addition, we identified key antitumor regulatory and metabolic pathways like nod-like receptor signaling and sphingolipid metabolism in KLF15-induced TNBC inhibition, broadening the range of potential KLF15 mediated tumor inhibiting pathways.

## Results

### KLF15 high expression in BrCa is associated with better patient survival

**Figure 1:**
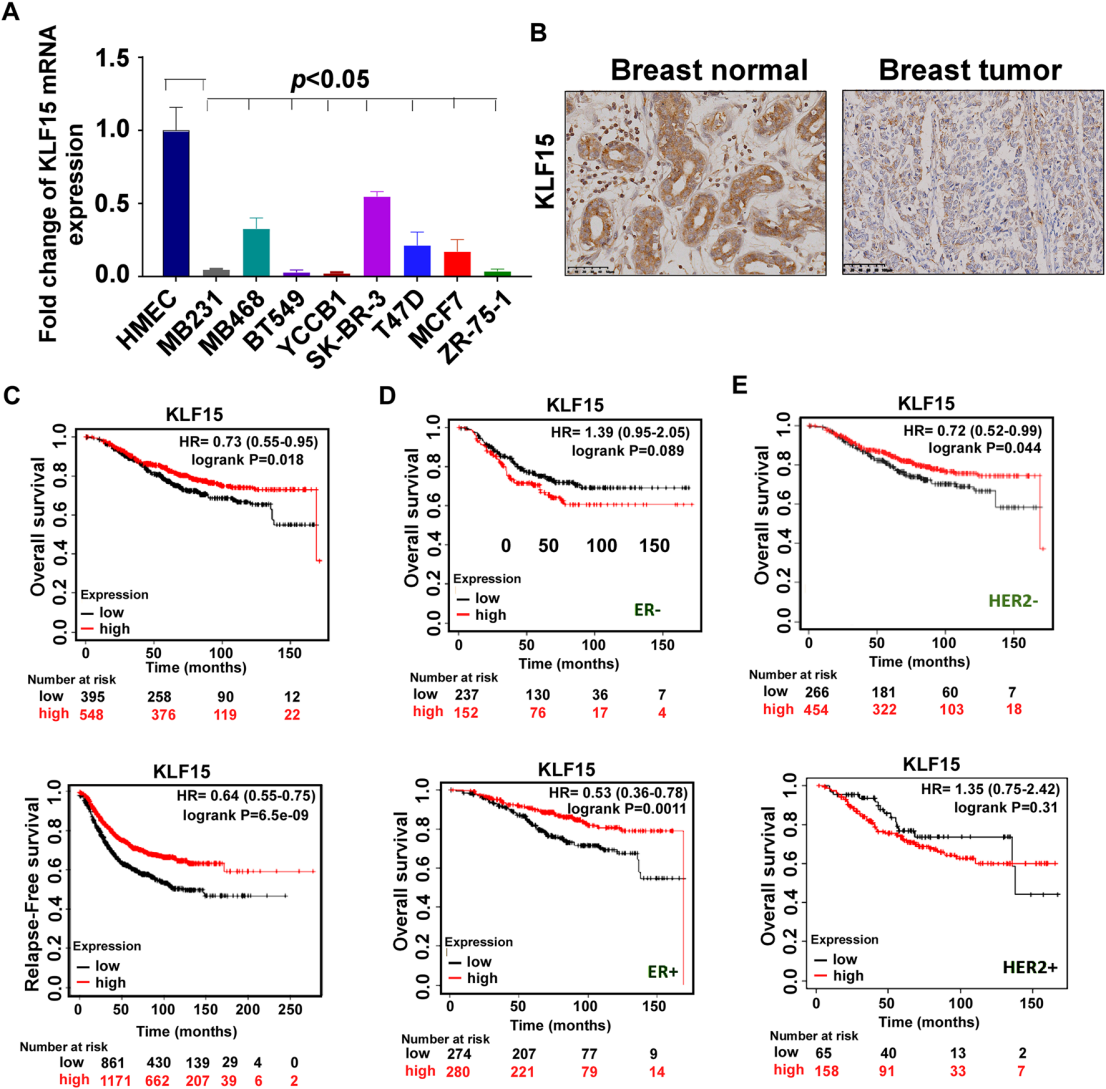
The expression levels of KLF15 in breast cell lines and TNBC tissues. (**A**) Quantitative real-time PCR (qPCR) analysis of KLF15 mRNA expression in different breast cancer cell lines. HMEC was used as non-tumoral control. (**B**) Representative images of KLF15 IHC staining in breast tumor and para-cancerous tissue. (**C**) High expression of KLF15 were beneficial to breast cancer patient prognosis. (**D**) The expression level of KLF15 was a protective factor in ER+ status patients, as opposed to a risk factor in ER− status. (**E**) The expression value of KLF15 was a risk factor in HER2+ status patients, but a protective factor in HER2− status.

To investigate KLF15 status in BrCa, we first used RT-qPCR assay to examine its expression in multiple BrCa cell lines and normal breast cell line HMEC. KLF15 expression was significantly lower in BrCa cell lines than in normal cell line (Fig. 1A). The KLF15 protein level was lower in breast tumor specimens compared to the para-cancerous tissues (Fig. 1B). Prognostic analysis from Kaplan–Meier Plotter (http://kmplot.com/analysis/index.php?p=service) showed that higher expression of KLF15 was associated with better patient survival (Fig. 1C). High KLF15 expression was also associated with better patient overall survival rates among ER+ patient, whereas the data suggested otherwise in ER− patients (Fig. 1D). However, high relative expression of KLF15 was correlated with poor overall survival rate in HER2+ patient, but the opposite in HER2− patient (Fig. 1E). Together, these data demonstrated downregulated KLF15 expression in BrCa. High levels of KLF15 can be regarded as an indicator of better prognosis broadly speaking, however the specific influence is dependent of pathologic types.

### Methylation of KLF15 promoter plays a critical role of its downregulation in BrCa

**Figure 2:**
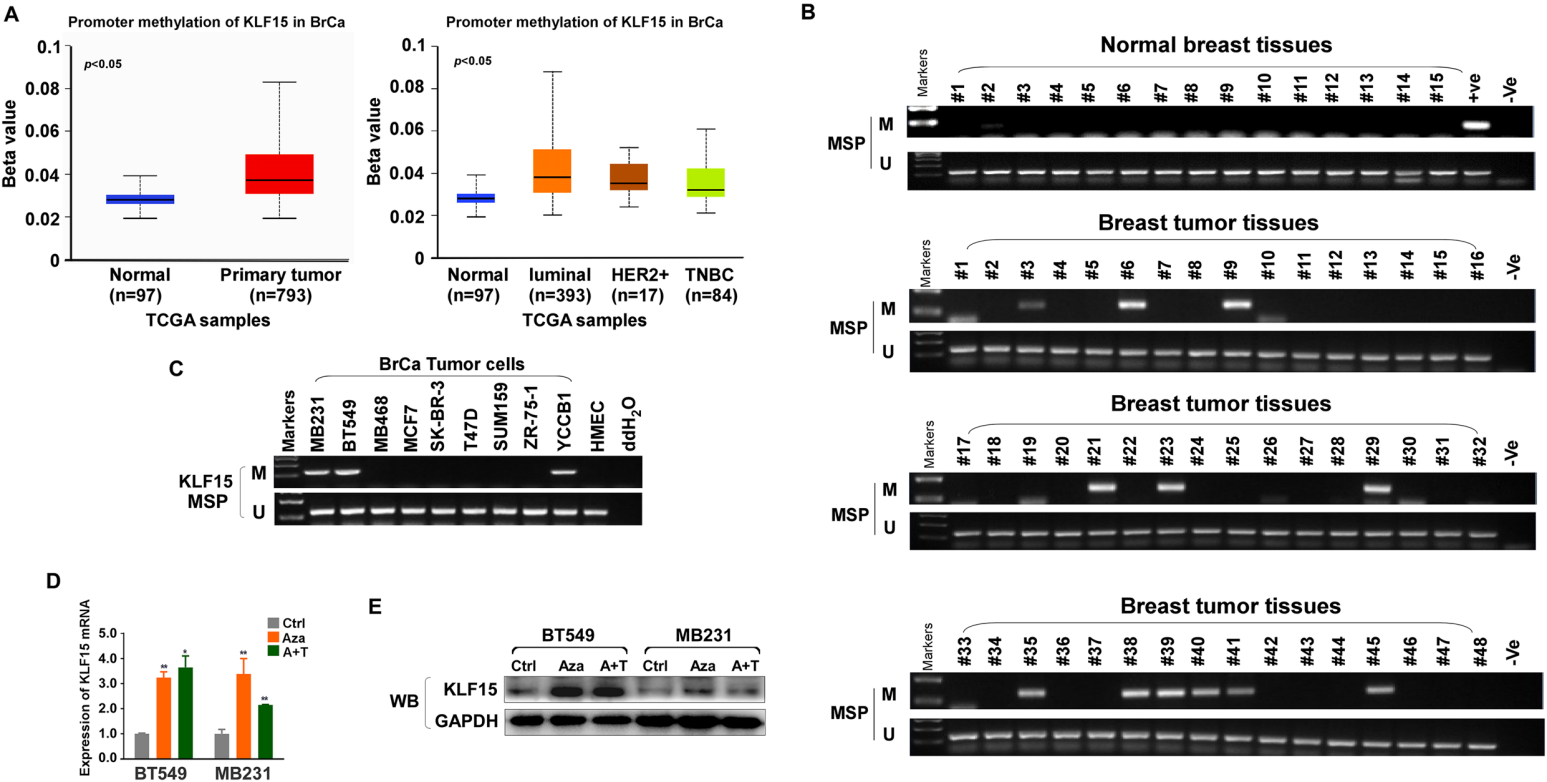
Methylation status of KLF15 promoter in breast carcinoma. (**A**) methylation status of KLF15 promoter in BRCA patients with data from TCGA samples. (**B**) Representative methylation of KLF15 in breast tumor and normal tissues as examined by MSP. (**C**) MSP analysis of KLF15 promoter methylation in breast cancer cell lines. (**D**) RT-qPCR results of KLF15 expression after treatment of Aza or Aza + TSA (A + T) in BT549 and MDA-MB231 cell lines. (**E**) Western blotting indicating KLF15 protein expression after Aza or A + T treatment in BT549 and MDA-MB231 cells.

DNA methylation is a key mechanism repressing the expression of certain tumor suppressor genes in cancer. Given low KLF15 expressions, we analyzed promoter methylation status of KLF15 among breast cancer patients with samples gathered from TCGA using online tools. Subtype is also considered. Higher levels of methylation can be observed among primary cancer patients (Fig. 2A). Further, we performed Methylation-specific PCR (MSP) analysis on 15 normal breast tissues and 192 breast tumor tissues. KLF15 promoter methylation was detected in 0/15 (0%) normal breast tissues while 25/192 (13%) tumor tissues (Fig. 2B). MSP analysis showed that the KLF15 promoter was methylated in 3/9 (about 30%) breast cancer cell lines (Fig. 2C). In order to build a more persuasive case, we performed demethylation experiments. Under the influence of demethylation drugs, Aza and TSA, KLF15 expression restored both in mRNA levels and protein levels (Fig. 2D,E), confirming methylation is one critical reason of low KLF15 expression in BT549 and MDA-MB231 cells.

These data indicated that promoter methylation is one critical factor leading to KLF15’s silence or down-regulation in BRCA. We also gathered data on KLF15 methylation and clinicopathological features among breast patients in Supplementary Table S1.

### Overexpression of KLF15 inhibits proliferation of TNBC cells

**Figure 3:**
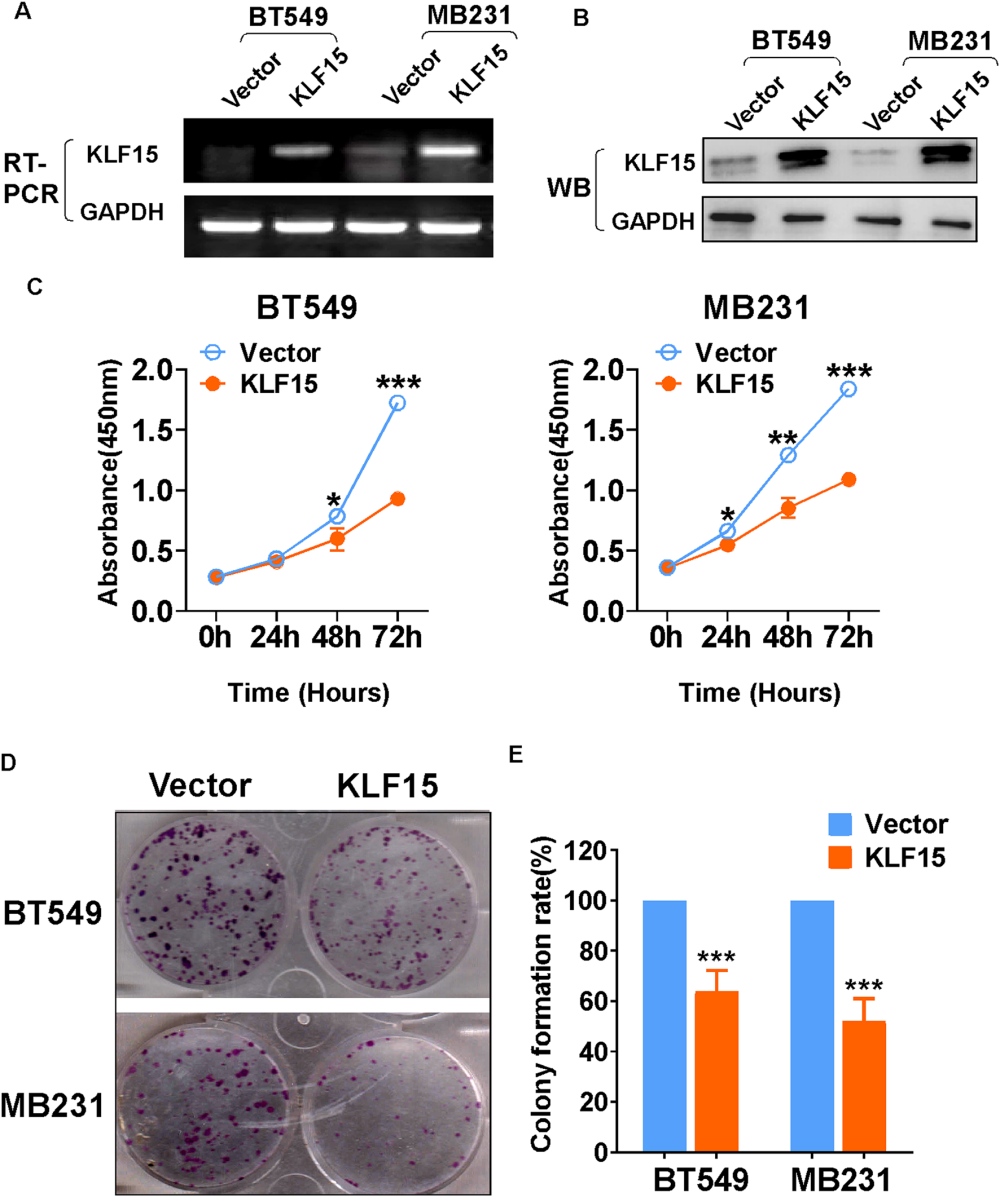
Overexpression of KLF15 inhibited the proliferation of TNBC breast cancer cells. (**A**,**B**) Validation of KLF15 overexpression by RT-PCR and Western blotting. (**C**) The capacity of cell proliferation was detected in MDA-MB231 and BT549 cells stably transfected with KLF15 or empty vector plasmid via CCK8 assay. (**D**,**E**) Representative images and histogram statistics of the colony-formation assay in vector- and KLF15-expressing MDA-MB231 and BT549 cells. *p < 0.05, **p < 0.01, ***p < 0.001. All experiments were performed in triplicate, respectively.

MDA-MB231 and BT549 were selected for a series of functional experiments in vitro*,* due to their relatively low expression among different breast cancers cell lines included. Restoration of KLF15 was completed through infection of KLF15 containing lentivirus selected by puromycin, and was confirmed by RT-PCR and Western blotting (Fig. 3A,B). Indicated by CCK8 assay, the proliferation rate of MDA-MB231 and BT549 cells was strongly inhibited after KLF15 overexpression (Fig. 3C). Compared with the empty vector group, MDA-MB231 and BT549 cells expressing KLF15 formed smaller and fewer colonies (Fig. 3D,E). In all, we managed to stably overexpress KLF15 in MDA-MB231 and BT549 cells, and verified its anti-proliferation ability in vitro.

### KLF15 induces cells cycle arrest and promotes apoptosis of TNBC cells

**Figure 4:**
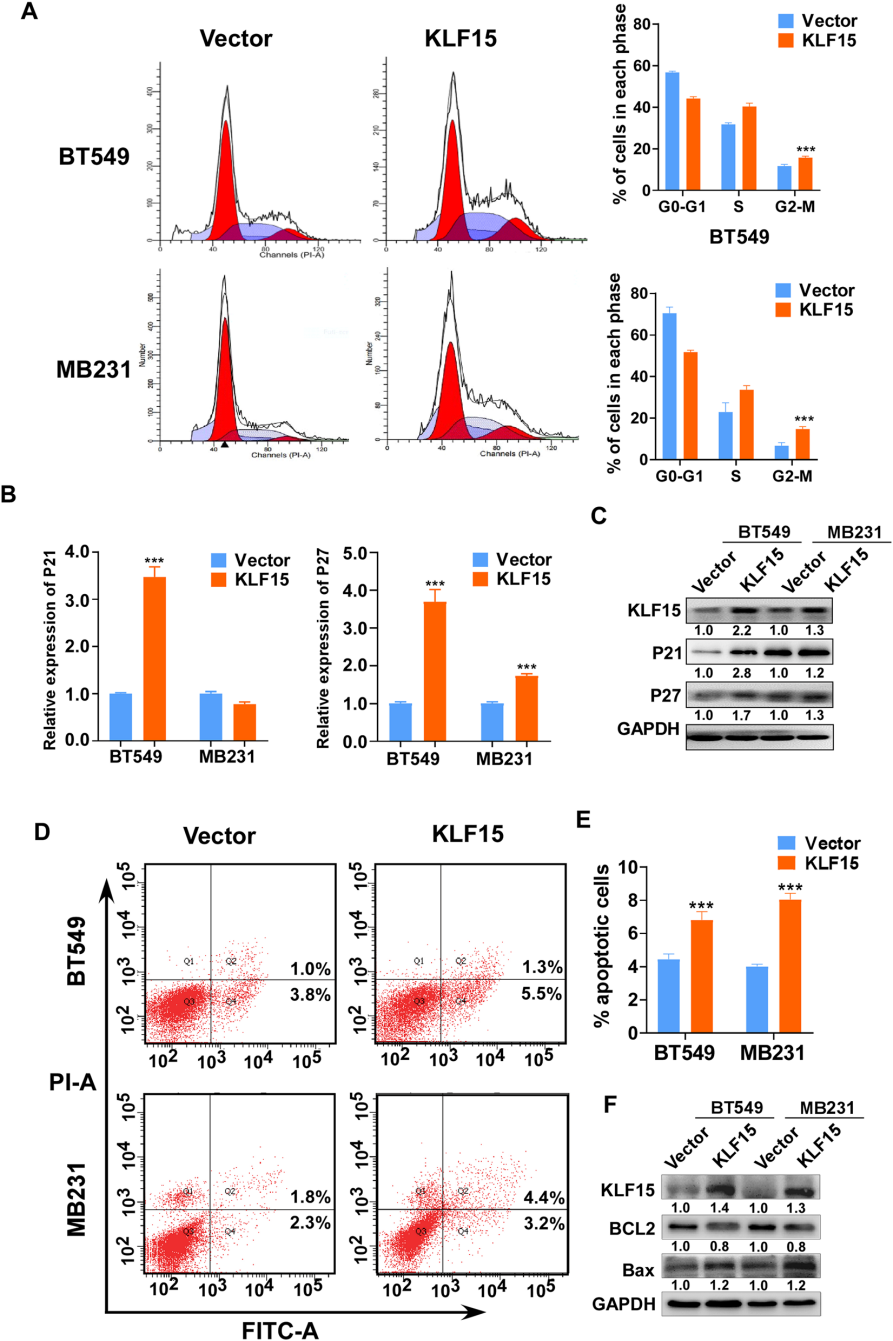
Exogenous KLF15 induces G2/M and S phase cell cycle arrest and promotes cell apoptosis in breast cancer cells. (**A**) Flow cytometry analysis was used to detect the effect of KLF15 on cell cycle in vector- and KLF15-expressing MDA-MB231 and BT549 cells. Left, representative flow cytometry results; right, histogram statistics of cell cycle changes. (**B**,**C**) RT-qPCR and Western blotting with primers and antibodies examining cell cycle associated regulators p21 and p27. (**D**,**E**) The proportion of apoptotic cells was detected in vector- and KLF15-expressing MDA-MB231 and BT549 cells by flow cytometry analysis. Representative flow cytometry plots and histogram statistics of apoptosis changes were exhibited. ***p < 0.001. (**F**) Western blotting detecting apoptosis related proteins, including Bcl-2 and Bax.

Cell cycle regulation also matters in carcinogenesis. We performed flow cytometry assay to investigate the effects of KLF15 has on cell cycle progression. The results showed that cells tend to accumulate at G2/M phase in KLF15-transfected cells in comparison of the vectors. Also, KLF15-overexpressed cells were arrested at S-phase in MDA-MB231 and BT549 cells (Fig. 4A). To confirm the cell cycle alteration, Western blotting and RT-qPCR were used to examine the expression of cell cycle-related factors p21 and p27. As the results implied, p21 and p27 were upregulated in both cells, whereas p21 were only discovered increased within KLF15-transfected BT549 cells (Fig. 4B,C).

Next, Annexin V-FITC/PI staining-based flow cytometry assays were conducted to value apoptosis changes. We observed that, apoptotic cell ratio increased in KLF15 overexpressing MDA-MB231 and BT549 cells, compared with the controls (Fig. 4D,E). Furthermore, Western blotting showed that exogenous expression of KLF15 downregulated anti-apoptotic protein Bcl-2 and enhanced the pro-apoptotic protein, Bax, in both cells (Fig. 4F). These results indicated that KLF15 induces G2/M and S phase cell cycle arrest and accelerates apoptosis in breast cancer cells.

### KLF15 suppresses migration and invasion ability of TNBC cells

**Figure 5:**
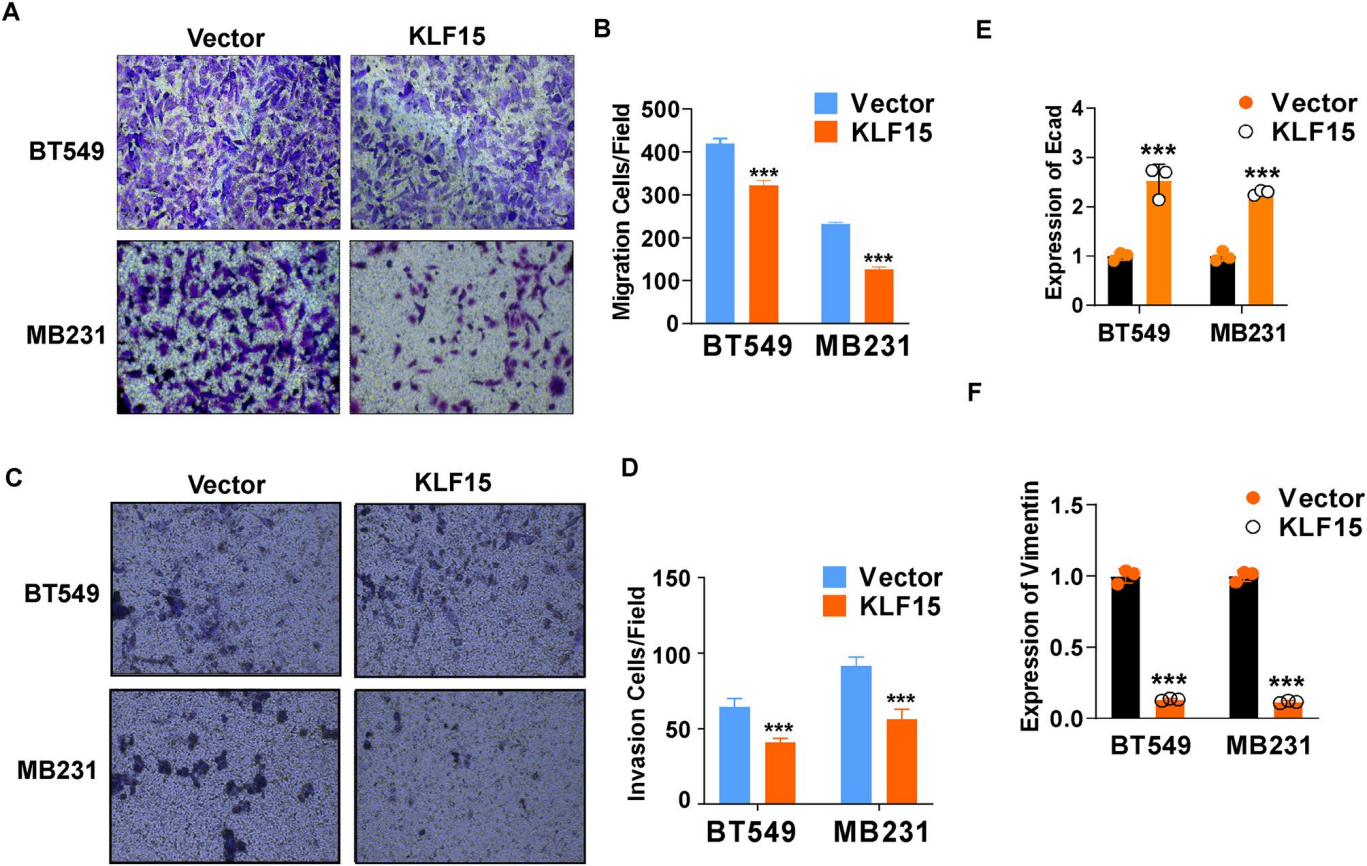
Overexpressed KLF15 suppressed breast cancer cell migration and invasion ability. (**A**,**B**) Representative images and quantification of Transwell migration assay conducted with stably transfected MDA-MB231 and BT549 cells, magnification times: × 200. (**C**,**D**) Representative images and quantification of Transwell invasion assay conducted with stably transfected MDA-MB231 and BT549 cells, magnification times: × 200. Three independent experiments were performed respectively. (**E**,**F**) After MDA-MB231 and BT549 cells were overexpressed with KLF15, mRNA expression of E-cadherin and Vimentin were examined. ***p < 0.001.

Up next, we intended to explore whether KLF15 can affect migration or invasion capacities of TNBC cells. Transwell experiment results indicated that exogenous expression of KLF15 significantly inhibited migration and invasion abilities of BT549 and MDA-MB231 cells (Fig. 5A–D). It has been widely known that Epithelial–Mesenchymal Transition (EMT) plays an important role in tumor cell metastasis^22^. Therefore, we detected EMT marker levels in KLF15 expressing cells and discovered that exogenous KLF15 caused an increase in E-cadherin while decreased the expression of Vimentin (Fig. 5E,F). These findings suggested that KLF15 has the capacity of inhibiting migration and invasion in vitro through reversing EMT process.

### KLF15 inhibits TNBC growth in vivo

To study the role KLF15 has on TNBC in vivo, a xenograft tumor model was established using nude mice. Slower tumor growing speed and smaller tumor size were observed in tumors derived from KLF15-overexpressing MDA-MB231 cells, as compared to control groups (Fig. S1A–C). The immune-histochemical assay suggested that the proliferation marker Ki67 expression level was significantly lower in the KLF15-expressing tumors (Fig. S1D). These data indicated that KLF15 can play an effective inhibitory role in breast cancer in vivo, consistent with the aforementioned results in vitro.

### KLF15 directly targets and downregulates CCL2 and CCL7 in TNBC cells

**Figure 6:**
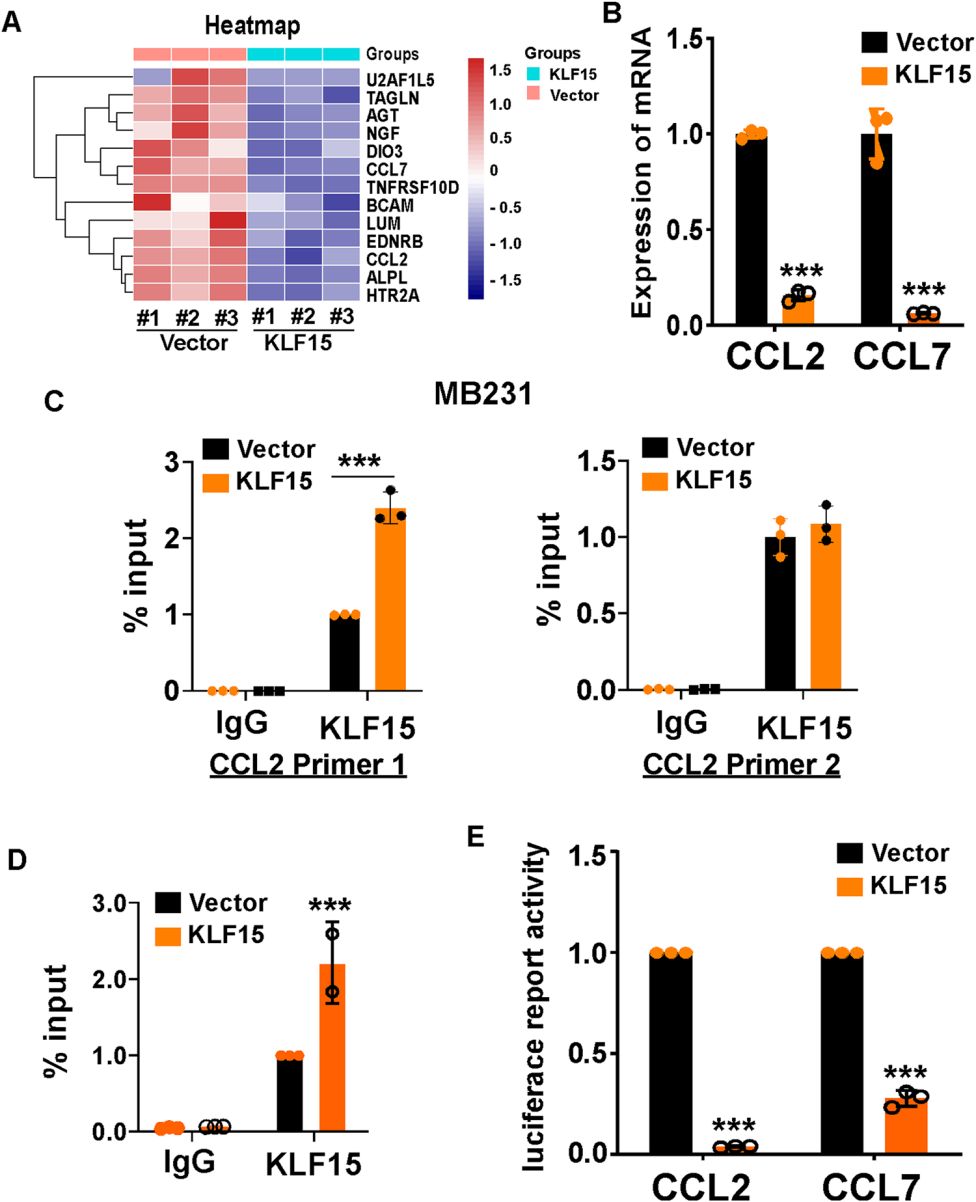
KLF15 decreases CCL2 and CCL7 expression by directly targeting and downregulating CCL2 and CCL7 in breast cancer cells. (**A**) Heatmap showing relative expression of transcripts altered by KLF15 overexpression in BT549 cells. (**B**) Expression of CCL2 and CCL7 were examined via RT-qPCR in BT549 cells transfected with vector or KLF15 plasmid. (**C**,**D**) ChIP analysis showing KLF15’s occupancy on CCL2 and CCL7 target promoters in MDA-MB231 cells. (**E**) Luciferase assay was performed to detect the transcriptional regulation of KLF15 at CCL2 and CCL7-3’ UTR in BT549 cells.

So far, the tumor suppressor identity of KLF15 in TNBC has been validated. Considering KLF15’s transcriptional factor role, RNA-seq was performed to analyze downstream regulated DNAs. As the results exhibited, a total of 133 genes were screened out in KLF15 overexpressed BT549 cells, among which 27 found downregulated. We focused on CCL2 and CCL7 for their relatively outstanding statistical significance (Fig. 6A). RT-qPCR was subsequently performed to confirm the down regulated mRNA levels of CCL2 and CCL7 in KLF15-expressing BT549 cells (Fig. 6B).

Subsequently, we concentrated on DNA binding ability of KLF15 upon targeted DNA. ChIP assay was used to ascertain the interaction between KLF15 protein and CCL2/CCL7 DNA along the genome. We observed elevated KLF15 binding at CCL2/CCL7 promoter sites in KLF15 overexpressed cells compared to cells from the control group (Fig. 6C,D). To determine whether there exists a regulative connection between KLF15 and CCL2/CCL7, dual luciferase reporter assay was performed. Potential KLF15 binding sites within the promoter of CCL2 and CCL7 were first identified using a bioinformatics website (http:// www. targetscan.org/). The pGL3-CCL2 and pGL3-CCL7 vector containing promoter sequences of CCL2 and CCL7 were used to validate the direct effect of KLF15 on CCL2 and CCL7 expression respectively (Fig. 6E). The above results showed that KLF15 directly binds and inhibits CCL2 and CCL7.

### Overexpression of CCL2 or CCL7 relieves proliferation and metastasis suppression by KLF15 in TNBC cells

**Figure 7:**
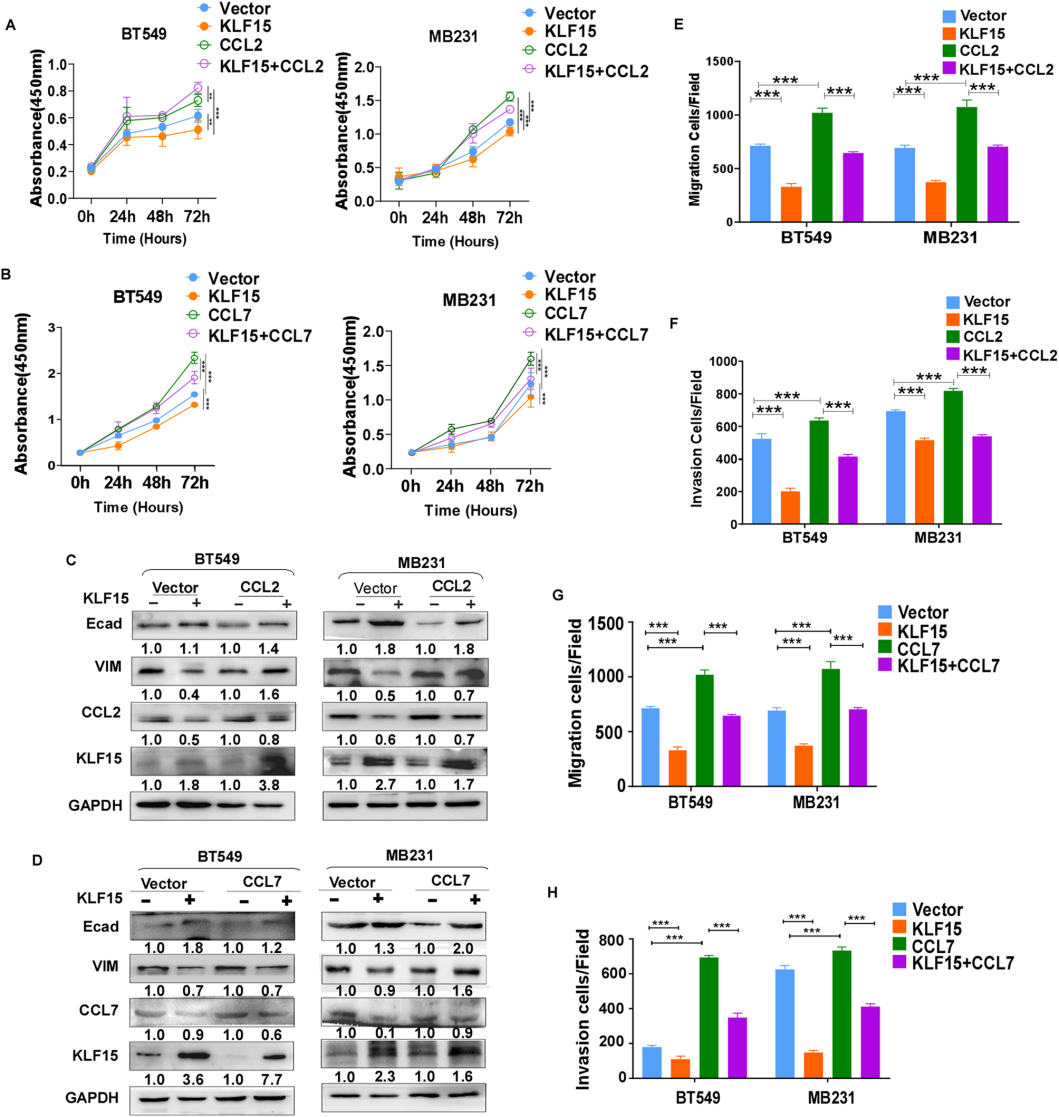
Exogenous CCL2 or CCL7 expression partially reverses KLF15’s effect on proliferation, migration and invasion. (**A**,**B**) Effect of acquired CCL2 or CCL7 overexpression on the proliferation of KLF15-overexpressing MDA-MB231 and BT549 cells. (**C**,**D**) Expression of CCL2 or CCL7 reverses EMT markers by KLF15. (**E**,**F**) Histograms showing migration and invasion results after CCL2 restoration in KLF15 expressing MDA-MB231 and BT549 cells. (**G**,**H**) Histograms showing migration and invasion results after CCL7 restoration in KLF15 expressing MDA-MB231 and BT549 cells. ***p < 0.001.

As reported above, KLF15 inhibits tumor behaviors, and downregulates CCL2 and CCL7, we were eager to find out whether the downregulation of CCL2 and CCL7 had effects on KLF15-mediated proliferation, migration and invasion suppression. MDA-MB231 and BT549 cells were transfected with empty vector, KLF15, CCL2/CCL7, and co-transfected with KLF15 and CCL2/CCL7 respectively. Co-transfection of CCL2/CCL7 and KLF15 regained proliferative activity in BT549 and MDA-MB231 cells (Fig. 7A,B). Meanwhile Western blotting revealed KLF15 dramatically suppressed the expression of CCL2 in vector group but partially suppressed CCL2 expression in co-expressed group. Regaining CCL2 can promote EMT potential, and E-cadherin and Vimentin were correspondingly rescued. So was the case with CCL7 (Fig. 7C,D). We further detected metastasis functions including migration and invasion, and discovered that restoration of CCL2 or CCL7 partially reversed the diminished metastasis abilities by overloaded KLF15 (Fig. 7E–H). These results concluded that KLF15 inhibits proliferation, migration and invasion through downregulating CCL2 and CCL7 in TNBC cells.

### Regulatory pathway analysis from transcriptome and metabolome data

**Figure 8:**
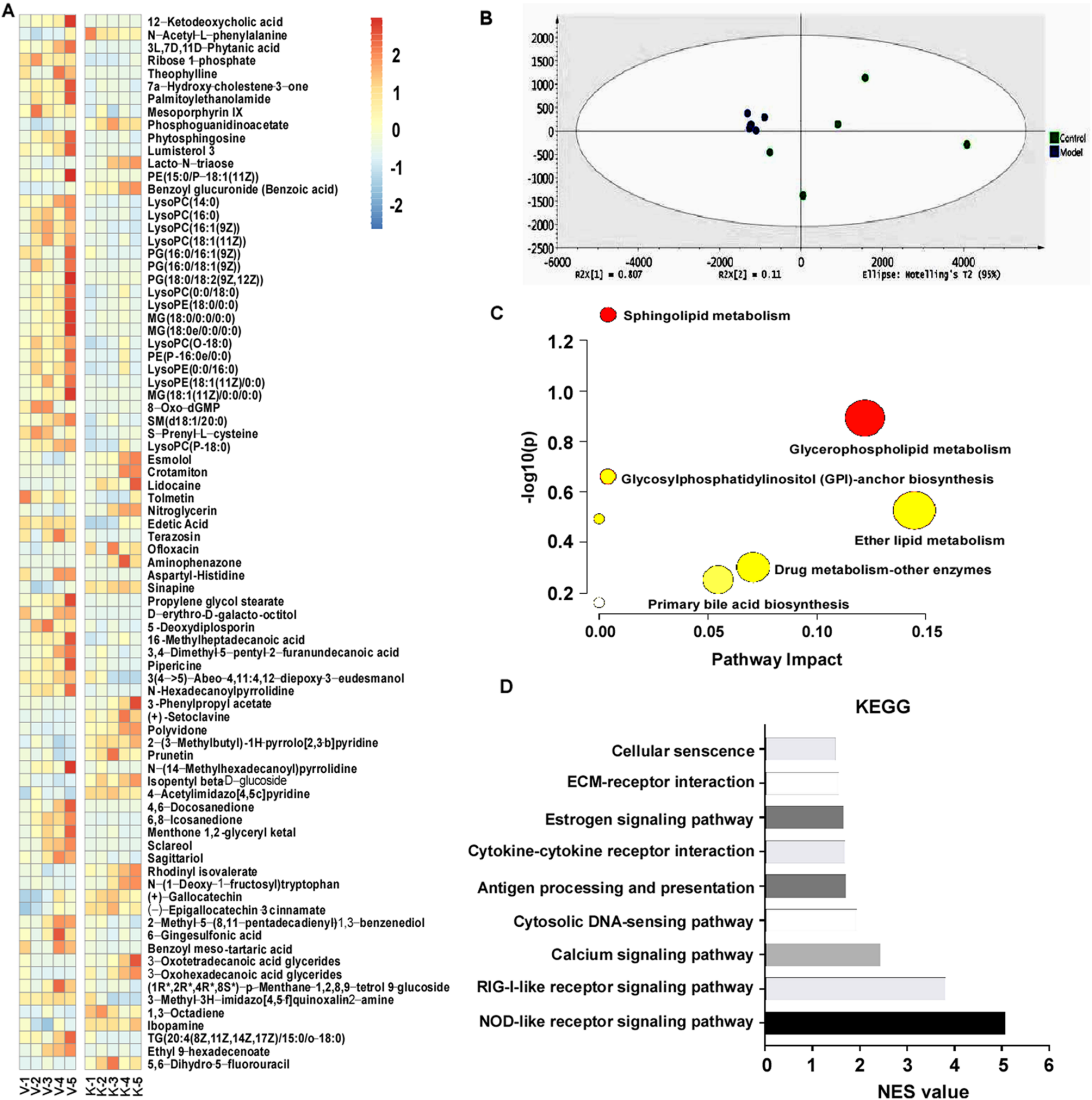
Metabolomic results and related bioinformatic analysis in KLF15-overexpressing BT549 cells. (**A**) Heatmap showing relative concentrations differences of metabolites within KLF15 and vector groups. With *p* < 0.05 and fold change > 2. V1–V5: Control group, K1–K5: Model group. (**B**) Score scatter plot of PCA analysis of the control and model groups based on metabolites with *p* < 0.05 and fold change > 2. (**C**) Pathway analysis of the metabolites with *p* < 0.05 and fold change > 2. D. KEGG pathway analysis of KLF15 regulation of antitumor pathways in breast cancer.

To take a further step looking into potential regulatory pathways of KLF15, we enlisted transcriptome and metabolome data. For RNA-seq, total RNA from KLF15 overexpressed BT549 cells was extracted and sent for sequencing. The transcriptomic sequencing results showed that, a total of 160 genes were influenced by KLF15, with 133 upregulated and 27 downregulated. We also collected metabolites from KLF15 and vector cells for metabolomic analysis. The results informed us of 82 differentially expressed metabolites (Fig. 8A). Score scatter plot of PCA analysis based on metabolites was also exhibited (Fig. 8B). Pathway analysis was carried out using MetaboAnalyst 5.0 (www.metaboanalyst.ca) bioinformatic online tool. Metabolic compounds with their corresponding concentrations were inputted for metabolic pathway enrichment analysis. KLF15 was suggestive of critical in many metabolic biological process. (Fig. 8C).

Finally, we took advantage of joint pathway analysis aiming to disclose major KLF15-driven regulatory pathways in TNBC based on the transcriptome and metabolome data. About 182 pathways were recognized, nine out of which were selected for deeper interpretation based on their significance in cancer biology (Fig. 8D)^23^. These pathways are nod-like receptor signaling pathway, rig-I-like receptor signaling pathway, calcium signaling pathway, cytosolic DNA-sensing pathway, antigen processing and presentation, cytokine-cytokine receptor interaction, estrogen signaling pathway, ECM-receptor interaction and cellular senescence. These findings suggested that KLF15 may exert its tumor-suppressor efficiency through modulating above mentioned pathways.

## Discussion

Previous investigation revealed that KLF15 is a potential tumor suppressor gene in breast cancer^15^, illustrating that KLF15 suppressed proliferation by inducing cell cycle arrest through upregulating p21 levels. However, important issues such as KLF15’s influence on proliferation and metastasis, its underlying antitumor signaling pathways and clinical significance in BrCa remain to be explained. This current study seeks to address above questions. In the present study, we collected enough data to demonstrate that KLF15 were able to inhibit TNBC growth both in vitro and in vivo. Promoter methylation is one critical modification of low or even silenced KLF15 expression in cell lines and patient samples. KLF15 exhibited an antitumor role in functions such as apoptosis, migration, invasion and cell cycle progress. It can be rightly concluded that KLF15 plays a crucial role in inhibiting tumor growth in TNBC patients. To further explore possible anti-oncogenic pathways of KLF15, RNA-seq assay was conducted and two downstream Chemokines, CCL2 and CCL7 were identified. Previous studies indicated that CCL2 and CCL7 are implicated in promoting proliferation and metastasis of BrCa^18–20^. These two chemokines have been reported as protective factor for tumor cells, making it a viable therapeutic target in chemotherapy^21,24^. Dual luciferase reporter assay and ChIP assay showed that KLF15 directly targets and downregulates the expression of CCL2 and CCL7. Western blotting and RT-qPCR confirmed that KLF15 inhibits the expression of CCL2 and CCL7. The expression of CCL2 and CCL7 significantly restored proliferation, migration and invasion limited by KLF15.

In addition, we used transcriptome and metabolome to explore the transcriptional and metabolic pathways. A total of 160 genes were found regulated by KLF15, with 133 upregulated while 27 downregulated. LC–MS metabolomics revealed 82 types of metabolites with varying concentrations. We then interpreted these data against the backdrop of the existing biological knowledge pertaining to how these entities are linked. Joint pathway analysis, done through MetaboAnalys5.0 bioinformatic tool, introduced 182 pathways, and 9 pathways were implicated in cancer biology. Data from this research provided abundant evidences suggesting KLF15 as an active participant in multiple antitumor pathways and a potential key player in regulating antitumor immune response.

CCL2 and CCL7, both highly expressed in tumor microenvironment, can recruit various immune cells (such as myeloid-derived suppressor cells, MDSCs) to form an immune-suppressive microenvironment, allowing tumor cell to evade from the body's immune surveillance and supporting tumor cell proliferation. The joint pathway analysis from the integration of transcriptome and metabolome data also showed that KLF15 is an active participant in onco-immune response pathways like nod-like receptor (NLR) signaling^25^, antigen processing and presentation^26^, rig-I-like receptor signaling, cytokine-cytokine receptor interaction and estrogen signaling. The other anti-tumor pathways regulated by KLF15 from joint pathway analysis are the cellular senescence signaling^27^, cytosolic DNA-sensing signaling^28^ and estrogen ecm-receptor interaction^29^.

KLF15 had the highest activity in nod-like receptor (NLRs) signaling pathway (*p* value, 4.87E-07). NES value from KEGG pathway analysis showed that KLF15 has a stronger relative activity in NLRs signaling pathway. NLRs have been established as crucial regulators in inflammation-associated tumorigenesis, angiogenesis, cancer cell stemness and chemoresistance^25^. In BrCa they play a crucial role in inhibiting tumor growth. Report by Correia et al. showed that NOD1 deficiency correlates with tumor growth and apoptosis failure in BrCa cells^30^. Overexpression of either NOD1 or NOD2 causes reduced cell proliferation and increased clonogenic potentials in BrCa cells^31^.

Metabolome data analysis derived from 82 metabolites revealed that KLF15 elevates the following metabolic pathways that have been implicated in a multitude of cancers including BrCa. They include sphingolipid metabolism, glycerophospholipid metabolism, glycosylphosphatidylinositol (GPI)-anchor biosynthesis and ether lipid metabolism. Sphingolipids regulate various biological processes such as growth, proliferation, and/or metastasis. Sphingolipids comprising of two central bioactive lipids, ceramide and sphingosine-1-phosphate (S1P), have opposing roles in regulating cancer cell death and survival by controlling signaling functions within the cancer cell signal transduction network^32^. Cellular stress caused by tumorigenesis induces the generation of sphingosine and ceramide through the activation of de novo synthesis pathways, sphingomyelin hydrolysis or the salvage pathway to mediate cancer cell death^33^. It was initially demonstrated that ceramide induces apoptosis in leukemic cells via coordinated activation of BAX and BAK^34,35^. Ceramides also play an important role in chemotherapy induced apoptosis^36,37^. Many studies suggest that sphingolipids also regulate the antitumor functions of various immune cell types. For example, ceramide inhibited the function of myeloid-derived suppressor cells (which normally inhibit cytotoxic T lymphocytes) by activation of lysosomal cathepsin B and cathepsin D, leading to attenuation of autophagy and induction of ER stress, therefore enhancing cytotoxic T lymphocyte function. Ether lipids (phospholipids) are expressed at much higher levels in cancer compared to normal cells. Despite the fact that proliferating and metastatic tumor cells require to accumulate and utilize lipids for their energy demands, there is considerable evidence that natural and semisynthetic antitumor ether phospholipids PNAE and PNAE(s) [plasmanyl-(*N*-acyl) ethanolamines] inhibit tumor growth^38,39^. These phospholipids have long been reported to have anti-inflammatory and immunomodulatory properties as natural healing agents^40^. In lymphoma studies, ether lipids have been reported to inhibit tumor by orchestrating apoptosis through ROS dependent manner^38^. Metabolomic analysis also revealed that KLF15 regulates drug metabolism in TNBC cells. Even though there are no current reports on KLF15 regulating chemo-metabolism in cancer, research work by Han S. and coworkers identified KLF15 as regulator of metabolism and elimination of hepatic endobiotics and xenobiotics (EXM) through various pathways^41^. They illustrated that KLF15 regulates all three phases (modification (phase I), conjugation (phase II), and excretion (phase III)) of the EXM system by direct and indirect pathways. This suggested that KLF15 might participate in key signaling pathway involved in anti-cancer drug metabolism in BrCa and might play important role in the potency and detoxification of anti-cancer drug administration to cancer patients.

In general, our study identified KLF15 as a tumor suppressor in TNBC, and illustrated that KLF15 suppresses TNBC proliferation and metastasis by targeting and downregulating CCL2 and CCL7. This study also discovered key antitumor regulatory pathways of KLF15, presenting an opportunity for detailed studies concentrating on the alternative directions of the antitumor activity of KLF15 in TNBC.

## Methods

### Cell lines and tissue samples

Nine BrCa cell lines (BT549, MDA-MB231, MCF7, T-47D, YCC-B1, ZR-75-1, MB468, SK-BR-3, SUM159) were obtained from American Type Culture Collection (ATCC, Manassas, VA). HMEC was used as non-tumoral control in breast cell lines. The cells were cultured in RPMI 1640 medium supplemented with 10% fetal bovine serum (FBS, Gibco-BRL) and 1% penicillin and streptomycin at 37 °C/5% CO_2_. HMEC cells were incubated in the DMEM medium (high glucose, HyClone, Logan, USA) with 10% FBS. All cell lines were either recently purchased or recently authenticated by STR profiling. Breast tissues were obtained from the Endocrine and Breast Surgery Department of the First Affiliated Hospital of Chongqing Medical University (Chongqing, China). Every sample was evaluated and subjected to histological diagnosis by expert pathologists. Every patient provided informed consent. All procedures for tissue collection were approved by the Institute Ethics Committee of the First Affiliated Hospital of Chongqing Medical University (approval notice 20150302). Samples were stored at the Chongqing Medical University tissue bank until used in the study.

### Reverse transcription PCR and quantitative real-time PCR

Reverse transcription was implemented using the Promega GoScript™ reverse transcriptase (Promega). Reverse transcription PCR (RT-PCR) was performed using the Go-Taq (Promega, Madison, WI, USA) and GeneAmp RNA PCR system (Applied Biosystems). GAPDH was used as internal control. SYBR Green (Thermo Fisher) and 7500 Real-Time PCR System (Applied Biosystems) were used to perform RT-qPCR. GAPDH was amplified as internal control. Primer sequences are listed in Supplementary Table S2.

### Bisulfite treatment, methylation-specific PCR(MSP) and de-methylation treatment

Genomic DNA was extracted from tissues and cell lines using the QIAamp DNA Mini Kit (Qiagen, Hilden, Germany). DNA bisulfite treatment was carried out according to previously published methods^42^. All primers were previously tested for their inability to amplify un-bisulfite DNA. PCR products were analyzed on 2% agarose gels. MSP primers for KLF15 are listed in Supplementary Table S2.

Aza and TSA were applied as de-methylation reagents for methylation inhibition. RT-qPCR and Western Blots were used to measure restored KLF15 on transcriptional and protein levels, separately. Online tools were taken advantage to analyze potential correlations between clinical pathological subtypes and KLF15 promoter methylation status.

### Construction of plasmids and stable cell lines

The KLF15-pEZ-Lv242, pCMV- CCL2 and pCMV-CCL7 plasmids were constructed by cloning the entire amplified coding region of KLF15, CCL2 and CCL7 into pEZ-Lv242 and pCMV respectively and sequenced for verification (GeneCopoeia, China). The promoter regions of CCL2 and CCL7 were cloned into pGL3-Basic vector. MDA-MB231 and BT549 cells were cultured in six-well plates. KLF15-pEZ-Lv242 lentivirus was then infected into 80% confluent MDA-MB231 and BT549 cells according to the manufacturer’s protocol. After, cells were grown in a non-selective growth medium for 48 h, after which it was replaced with a selection medium containing 24 μL/mL (MDA-MB231) and 10 μL/mL (BT549) puromycin (50 mg/mL) for 14 days. Overexpression of KLF15 was confirmed by Western blotting and quantitative real time PCR (RT-qPCR).

### Cell proliferation and colony formation assay

BT549-KLF15/vector and MDA-MB231-KLF15/vector cells were cultured in 96-well plates at a density of 2000 cells per well. Cell proliferation was evaluated at 0, 24, 48, or 72 h with the Cell Counting Kit-8 Assay (MTT, Promega) according to the manufacturer’s instructions. Absorbance values were measured at 450 nm employing the microplate reader (Multiskan MK3, Thermo Fisher Scientific, Schwerte, Germany). Colony formation assay was performed to detect cell proliferation. All experiments were performed in triplicate.

### Flow cytometry analyses of cell cycle and apoptosis

For cell cycle analysis, cells were digested with trypsin and fixed with ice-cold 70% ethanol, treated with 5 mg/mL RNase A (Sigma), stained with propidium iodide, and analyzed by flow cytometry (FACSCalibur instrument and CELLQUEST software, Becton Dickinson). For the apoptosis assays, cells were stained with annexinV-fluorescein isothiocyanate and PI (propidium iodide). Apoptosis and cell cycle status were analyzed using the CELL Quest software (BD Biosciences, San Jose, CA, USA). All experiments were performed in triplicate.

### Transwell migration and invasion assay

Transwell chamber inserts with 8-μm pores and coated with 70 μL of Matrigel (2.5 mg/mL; BD Biosciences Discovery Labware, 1:7 dilution) were used for the invasion assay. 3 × 10^5^ cells were seeded into the upper wells of pre-coated Transwell chambers. Lower wells of the Transwell chambers were filled with 700 μL of the same medium with 20% FBS. After a 48 h incubation, cells were fixed with 4% paraformaldehyde for 30 min and then stained for 30 min with Crystal violet. Photos of the cells that had migrated to the lower side of the filter were obtained using light microscopy. All experiments were repeated three times.

### Tumor xenograft model in nude mice

Tumor xenografts model in nude mice was conducted as previously described^43^. KLF15 and Vector expressing MDA-MB231 cells (4 × 10^6^ resuspended in 0.15 mL PBS) were injected subcutaneously into the right and left sides of BALB/c nude mice aged 4–6 weeks, respectively (n = 6). All procedures for tumor model construction were approved by the Institute Ethics Committee of the First Affiliated Hospital of Chongqing Medical University (approval notice 20150302). The authors all complied with the ARRIVE guidelines. All methods were performed in accordance with the relevant guidelines and regulations.

### Western blotting

Cells were lysed using a protein extraction reagent (Thermo Scientific, Rockford, IL, USA) containing protease inhibitor phenyl methane sulfonyl fluoride and phosphatase inhibitor cocktail (Sigma, St. Louis, MO, USA). Total protein concentrations were measured using the BCA protein assay reagent (Thermo Scientific, Rockford, IL, USA). The primary antibodies were used as follows: KLF15 (#sc-271675, Santa Cruz), CCL2 (#sc-32771, Santa Cruz Technology), CCL7 (#MA5-29089, Invitrogen), p21 (#2947, Cell Signaling Technology), p27 (#3686, Cell Signaling Technology), Bcl-2 (#2870, Cell Signaling Technology), Bax (#5023, Cell Signaling Technology), E-cadherin (#14472, Cell Signaling Technology), Vimentin (#5741, Cell Signaling Technology). GAPDH (#sc-47724, Santa Cruz Biotechnology) served as a loading control. Dilution ratio of primary antibody conducted in this section was 1:1000. Secondary antibody was purchased from biosharp (BL001A, BL003A, Biosharp, China). Photographic results of the blots were obtained using VILBER Fusion FX5.

To see the original blots please refer to the supplementary files. The membranes were cropped for the purpose of cost saving, and therefore there might be some absence of images of adequate length.

### Immunohistochemistry

Standard streptavidin–peroxidase immunohistochemistry was performed using the UltraSensitive TM SP Kit (Maixin-Bio, Fujian, China) according to the manufacturer’s instructions. Sections were dewaxed, rehydrated blocked, and then incubated with primary antibodies against KLF15 (1:100 dilution, #sc-271675) and Ki67 (1:100 dilution, #ARG53222, Arigo). The sections were then treated with a secondary antibody and stained with diaminobenzidine. Images were scanned and processed by KFBIO digital pathology slide scanner (Zhejiang, China).

### Dual-luciferase reporter assay

To verify whether CCL2 and CCL7 is a direct target of KLF15, target reporter plasmid containing wild type or mutant CCL2/CCL7 promoter sequence was constructed. MDA-MB231 and BT549 cells were seeded in 24-well plates and co-transfected with reporter plasmid according to the manufacturer's instructions. Luciferase activity was measured with a dual-luciferase reporter assay kit (Promega) after 48 h.

### Chromatin immunoprecipitation (ChIP) assays

The chromatin immunoprecipitation (ChIP) assay was performed according to the manufacturer’s instructions (Beyotime Institute of Biotechnology, Hangzhou, ZJ, China). The KLF15 antibody was used to precipitate the protein-DNA complexes, and the DNA isolated through the ChIP reactions was subjected to PCR using primers specific to the promoters of CCL2 and CCL7 respectively. Primers used were listed in Supplementary Table S2.

### RNA sequencing analysis

RNA-Sequencing libraries were generated using NEBNext® UltraTM RNA Library Prep Kit for Illumina® (NEB, USA) following manufacturer’s recommendations. A total amount of 3 µg RNA per sample was used as input material for the construction of cDNA libraries, whose quality was assessed on the Agilent Bioanalyzer 2100 system. Genes with an adjusted *p* < 0.05 and identified with DESeq^44^ were considered to be differentially expressed.

### Untargeted metabolomics (liquid-chromatography mass spectrometry)

5 μL of metabolite extracted from MDA-MB231 cells was loaded into the system for metabolomic fingerprinting. An ACQUITY I Class UPLC system (Waters, Milford, MA, USA), a Waters ACQUITY UPLC HSS T3 column (2.1 × 100 mm, 1.8 μm) and a Waters ACQUITY UPLC HSS T3 VanGuard™ precolumn (2.1 × 5 mm, 1.8 μm) were collectively applied for separating metabolites. The UPLC system was coupled with a G2-S QTOF system (Waters, Milford, MA, USA) in MS^E^ mode. The identification of different metabolites was conducted through Progenesis QI™ (Waters, Milford, MA, USA) and HMDB databases. Only samples with a mass accuracy within ± 5 ppm and overall score 36 were regarded as highly confident and saved for subsequent analysis. In total 5 replicates were sent for detection.

### Statistical analysis

Statistical analyses were performed using GraphPad Prism 9.0 software, KEGG, MetaboAnalyst 5.0 software and R software. Two tailed Student’s *t* test was used to evaluate the experimental results. The chi-square test was also used according to the specific. *p* values of all tests less than 0.05 was considered statistically significant.

### Ethics approval statement

All procedures for harvesting human tissue specimens were approved by the Institute Ethics Committee of the First Affiliated Hospital of Chongqing Medical University. The animal experiment conducted in this study is strictly abided by the Institute Ethics Committee of the First Affiliated Hospital of Chongqing Medical University with approval notice.

## Supplementary Information

Supplementary Information.

## Data Availability

Raw RNA-Sequencing data can be visited and obtained through https://github.com/lexin0099/RNA-sequencing-for-KLF15. The data source supporting the prognosis conclusions are available in the Kaplan–Meier Plotter (http://kmplot.com/analysis/index.php?p=service). Request for other materials should be addressed to corresponding author T.X. upon reasonable request.
