# Supplementary material for: KLF15 suppresses tumor growth and metastasis in Triple-Negative Breast Cancer by downregulating CCL2 and CCL7
Source: Sci Rep. 2022 Nov 8;12:19026. doi: 10.1038/s41598-022-23750-4 (PMC9643362; doi:10.1038/s41598-022-23750-4)
Supplement: Supplementary file 1 — Supplementary Information. [file 41598_2022_23750_MOESM1_ESM.pdf]

Supplementary Information

- **KLF15 suppresses tumor growth and metastasis in Triple-Negative Breast Cancer by downregulating CCL2 and CCL7**

- Quist Kanyomse[1], Xin Le[1], Jun Tang[2], Fengsheng Dai[2], Youchaou Mobet[3], Chang Chen[3], Zhaobo Cheng[2], Chaoqun Deng[2], Yijiao Ning[2],Renjie Yu[2], Xiaohua Zeng[4], Tingxiu Xiang[1,4,*]

Original gel images-Fig 2B

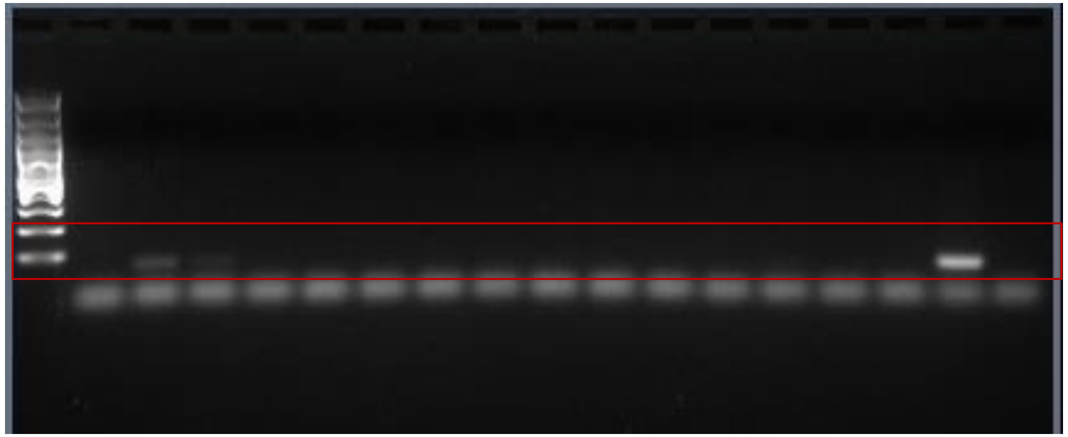

MSP-M

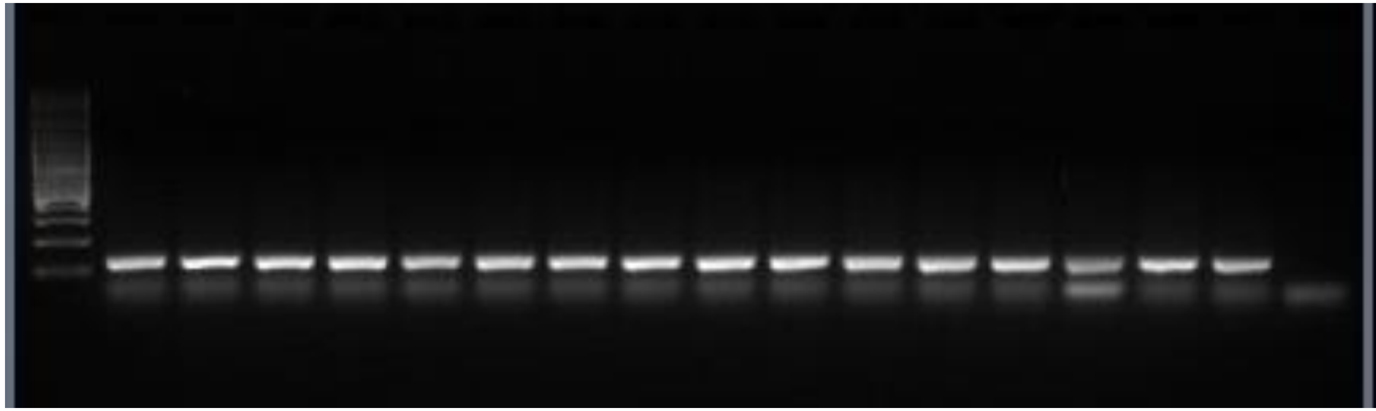

MSP-U

First Row

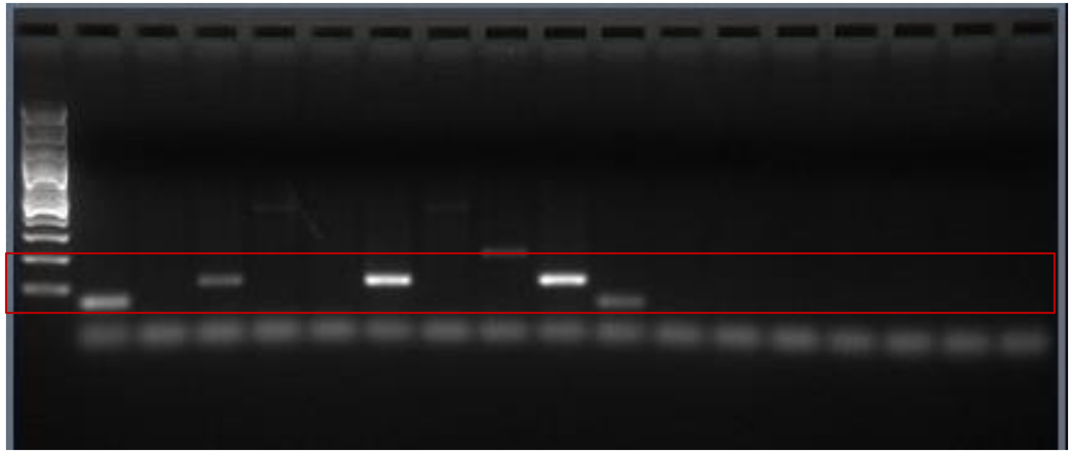

MSP-M

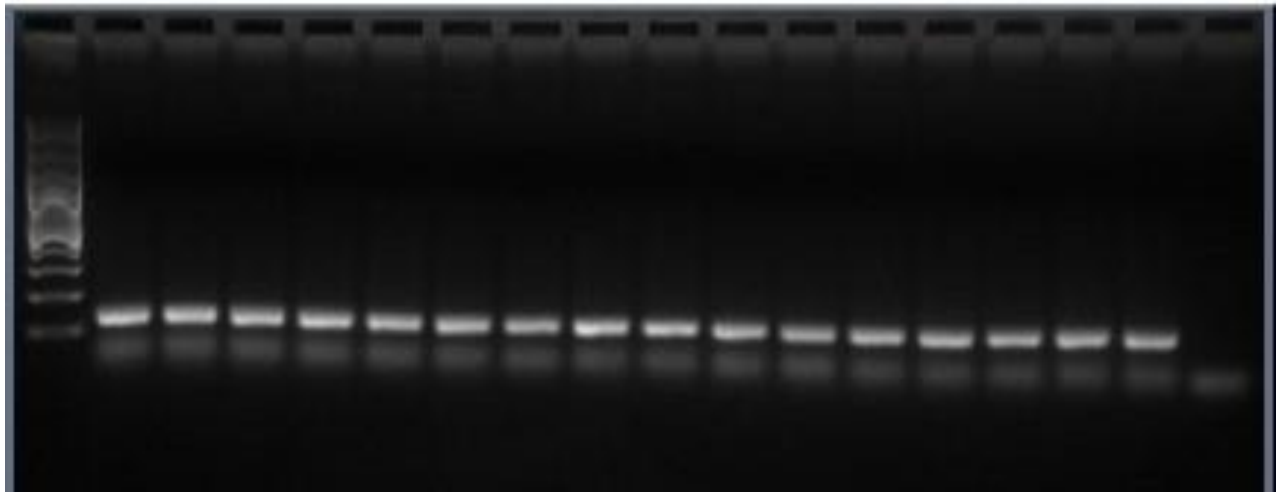

MSP-U

Second Row

Original gel images-Fig 2B

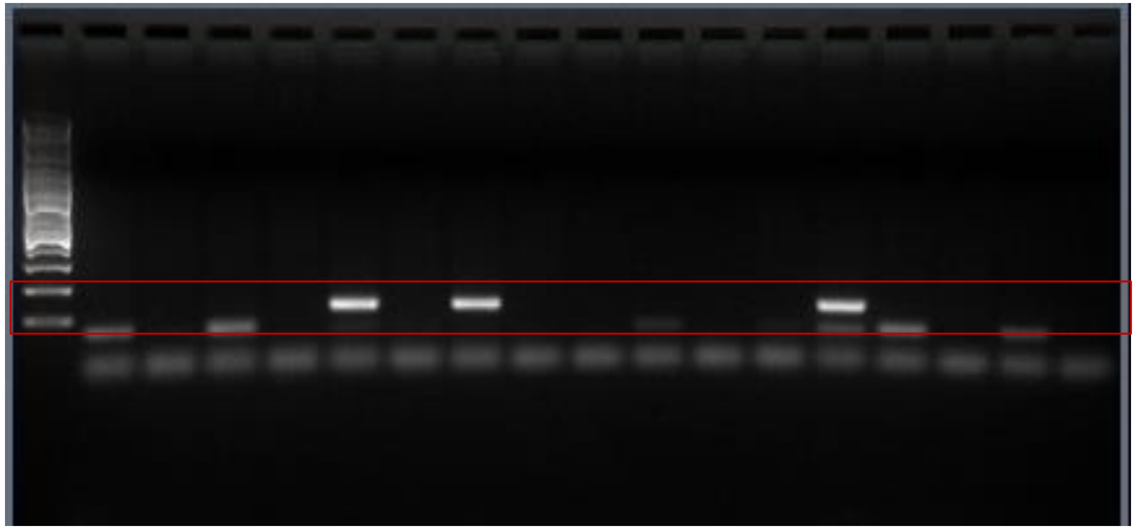

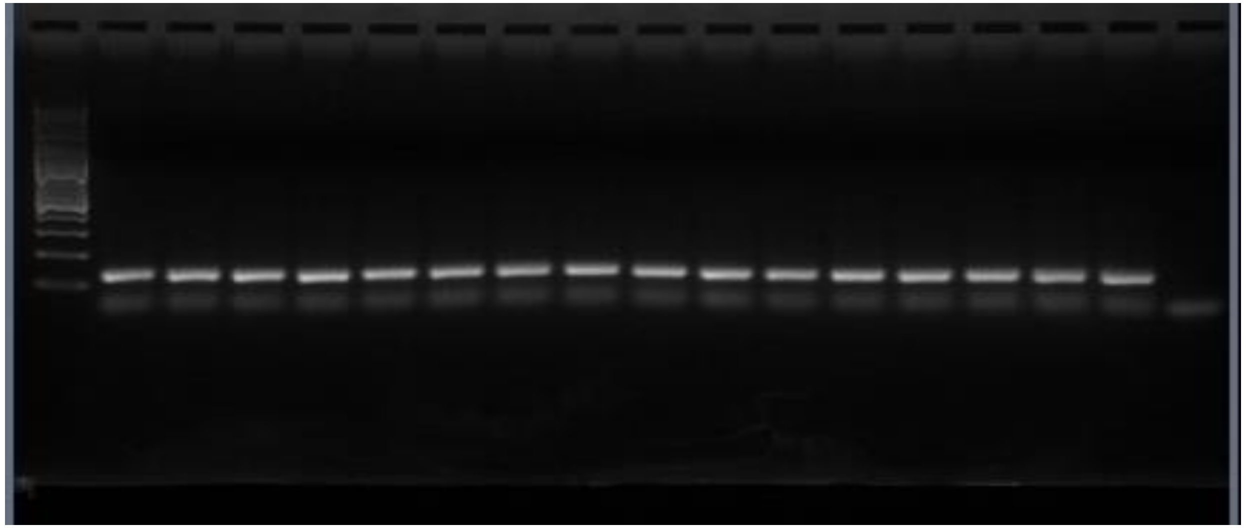

MSP-M **Third Row** **MSP-U**

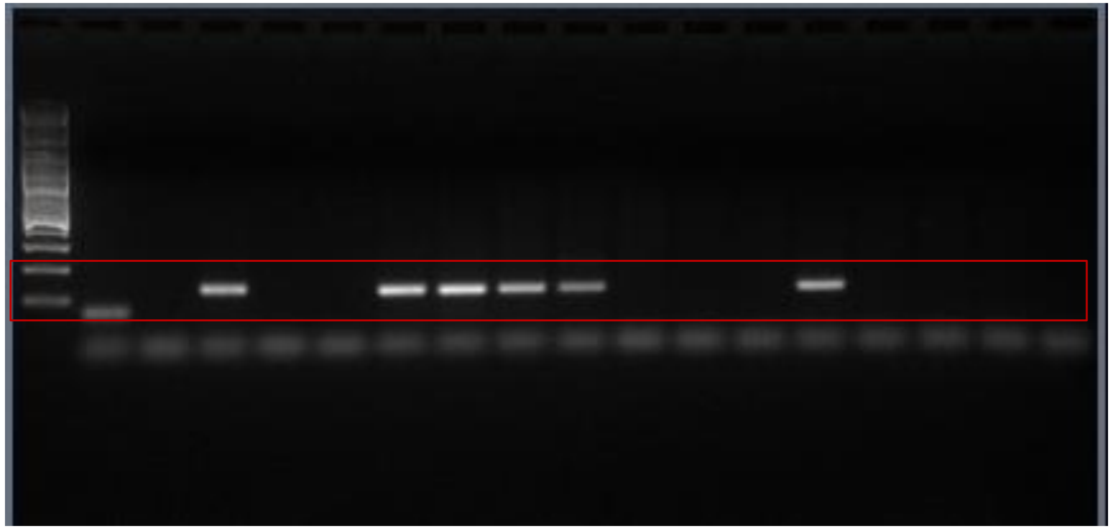

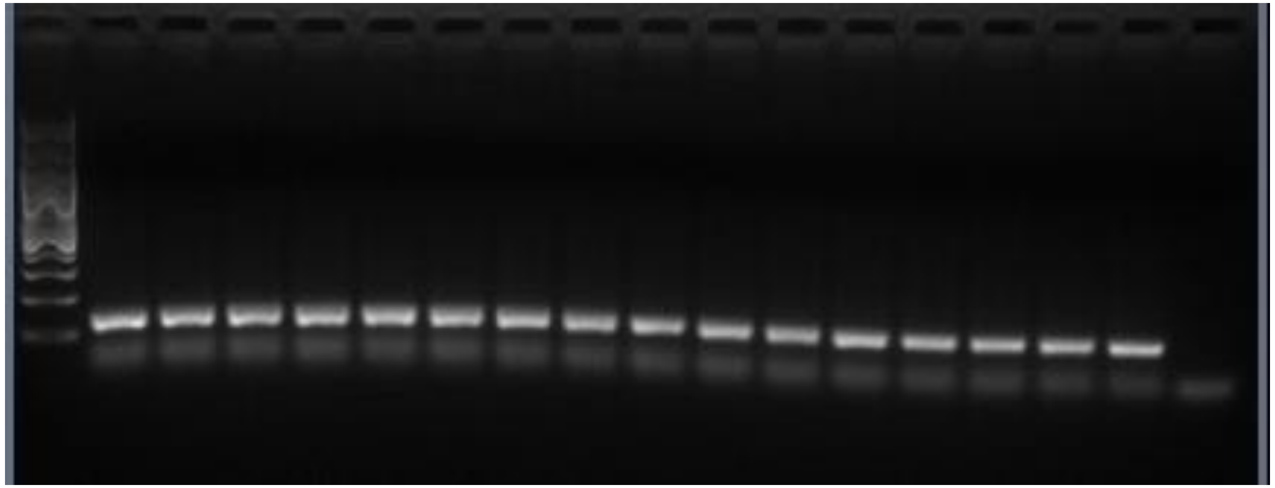

MSP-M **Last Row** **MSP-U**

Original gel images-Fig 2C

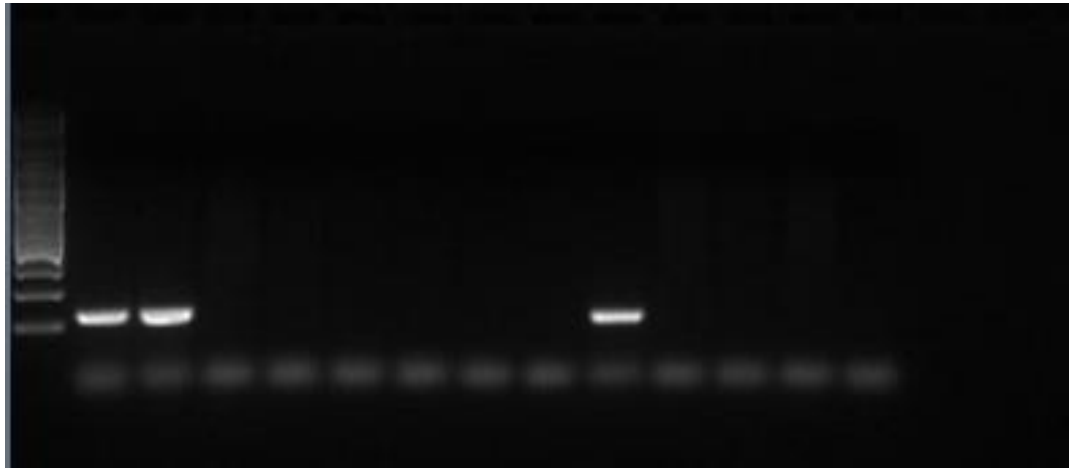

KLF15 MSP-M

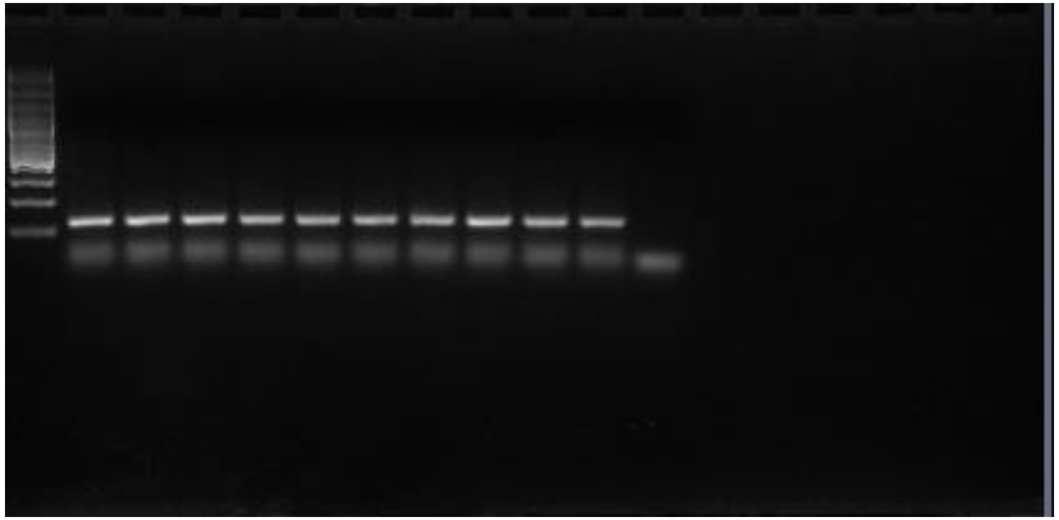

KLF15 MSP-U

Original Western blots images-Fig 2E

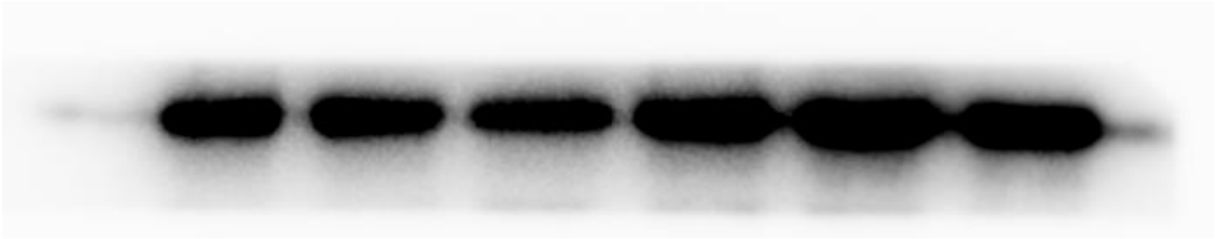

GAPDH protein

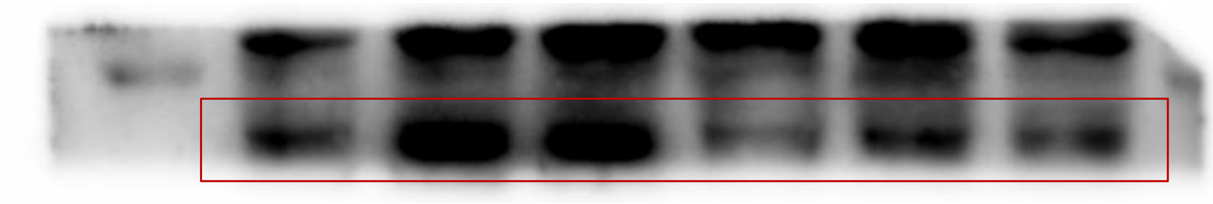

KLF15 protein

Original gel images-Fig 3A

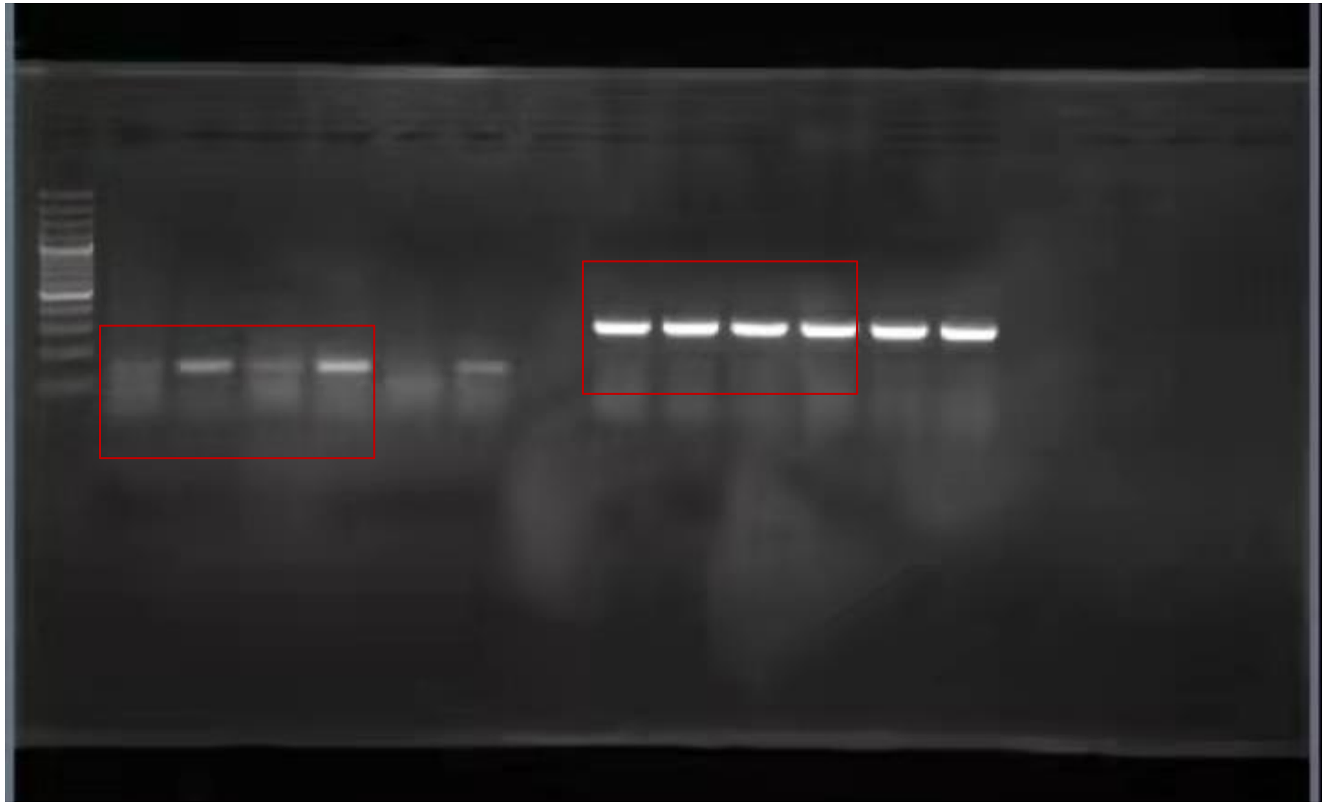

Original Western blots images-Fig 3B

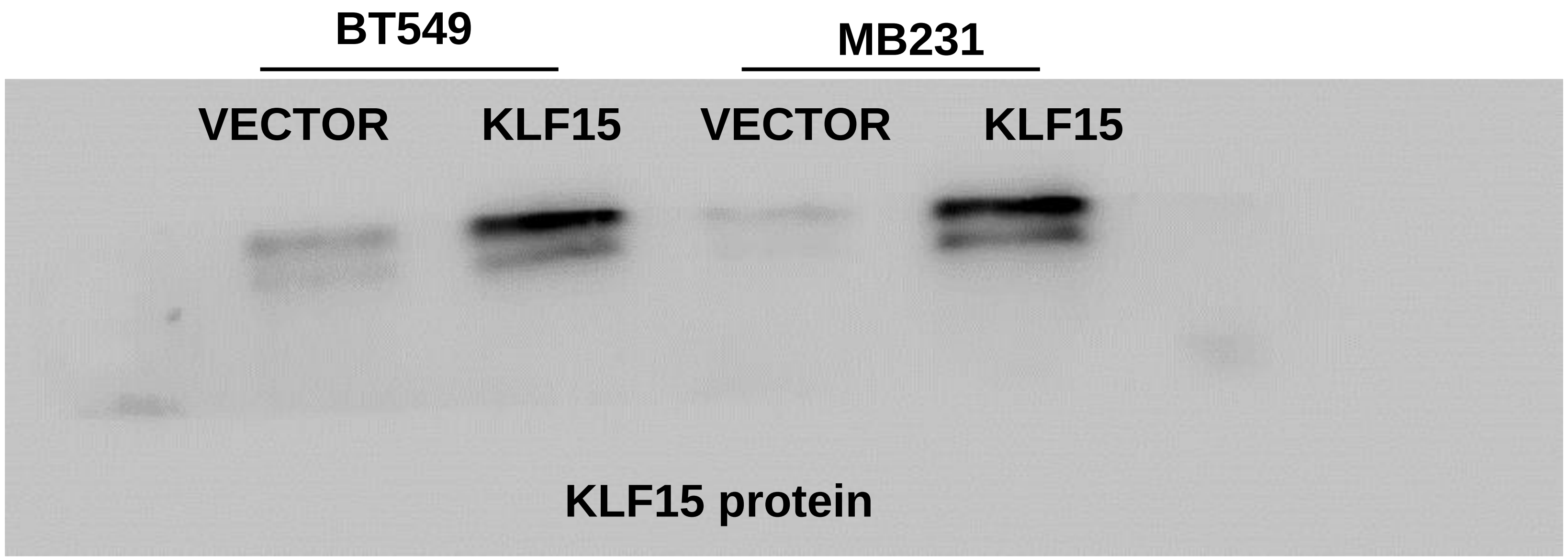

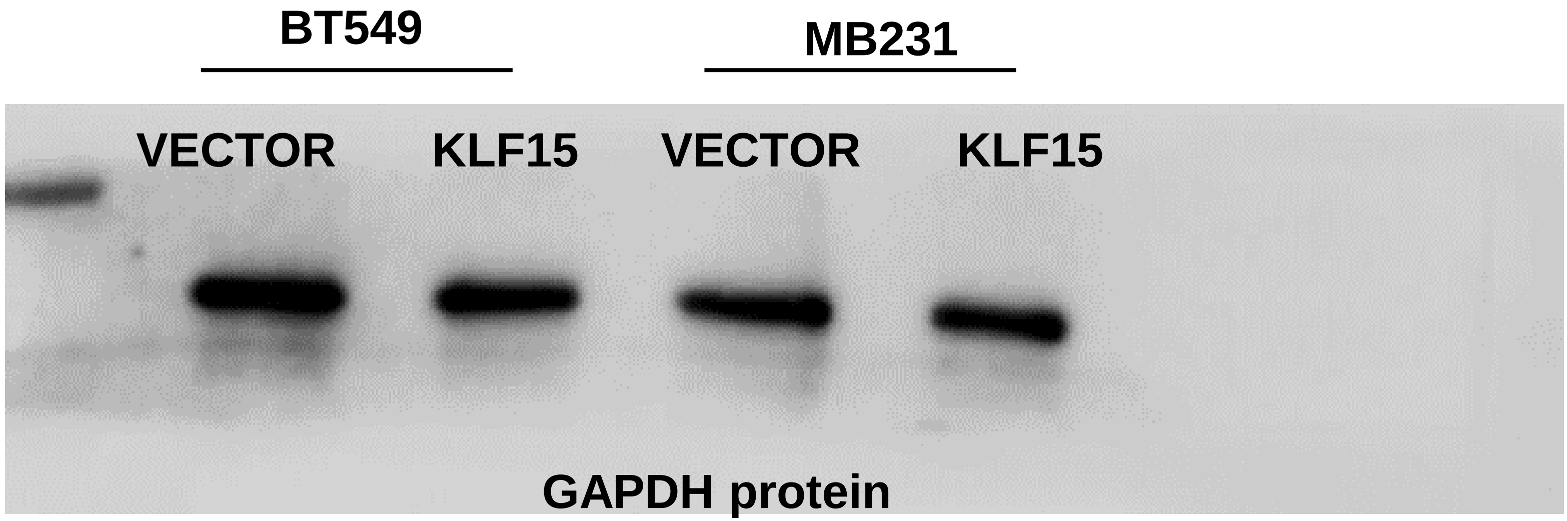

Original Western blots images-Fig 4C

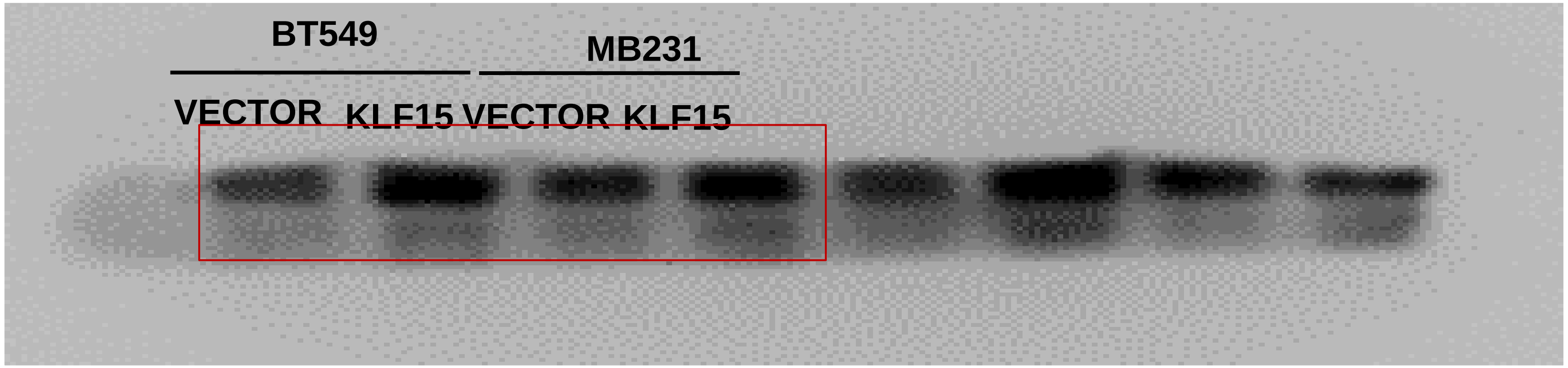

KLF15 protein

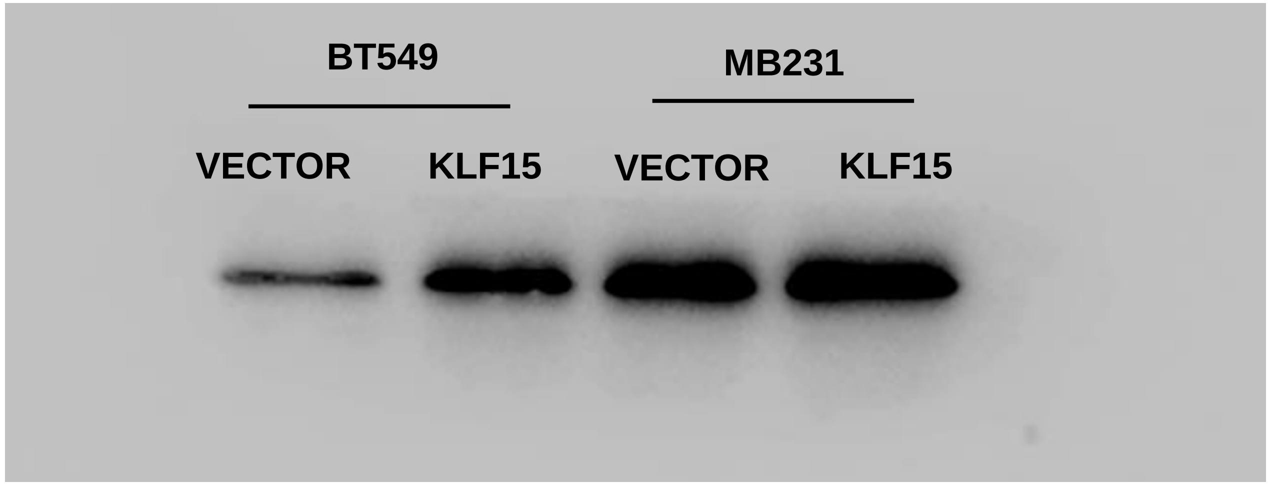

p21 protein

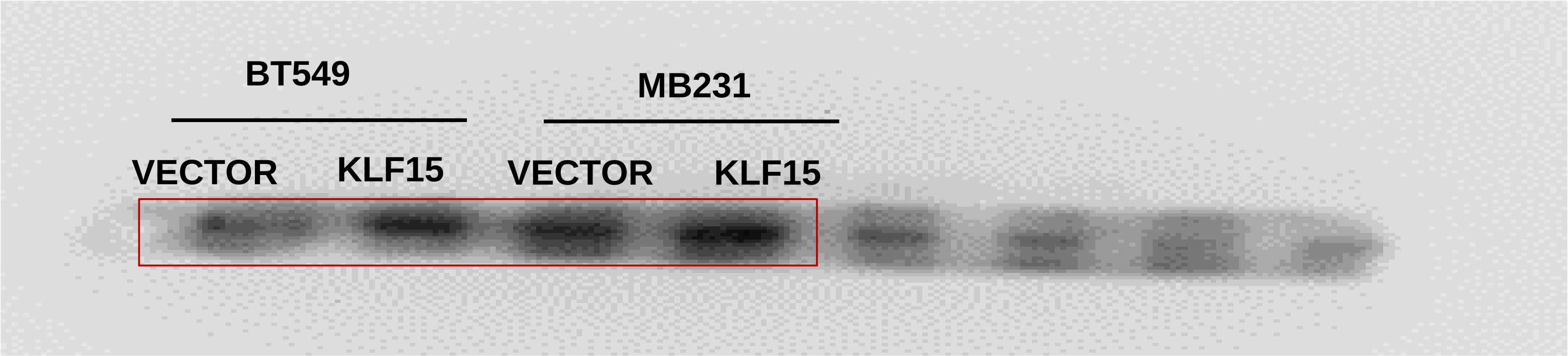

p27 protein

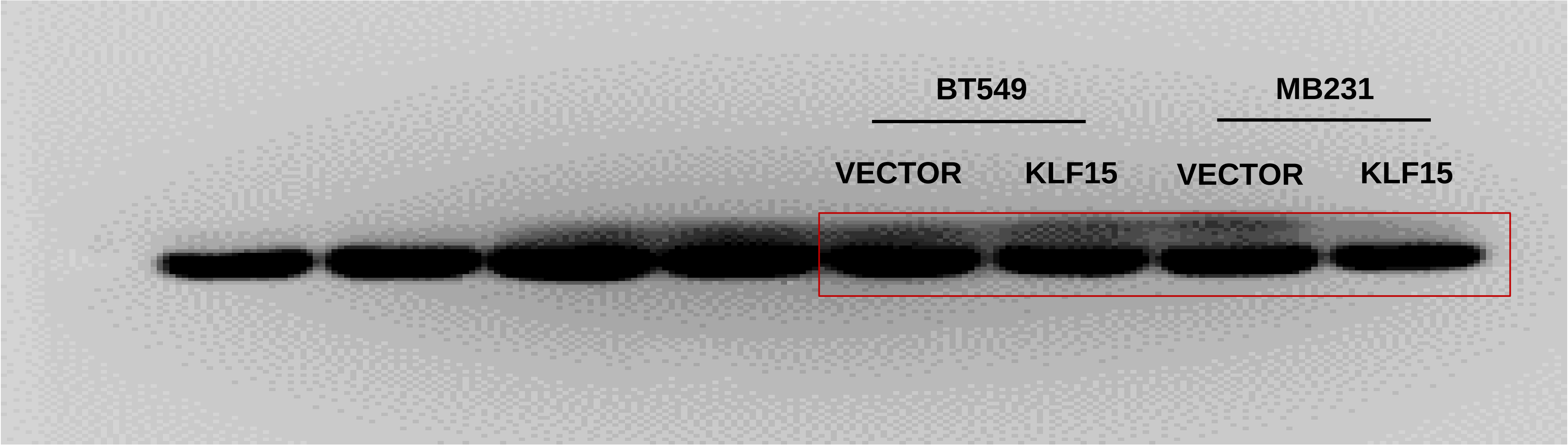

GAPDH protein

Original Western blots images-Fig 4F

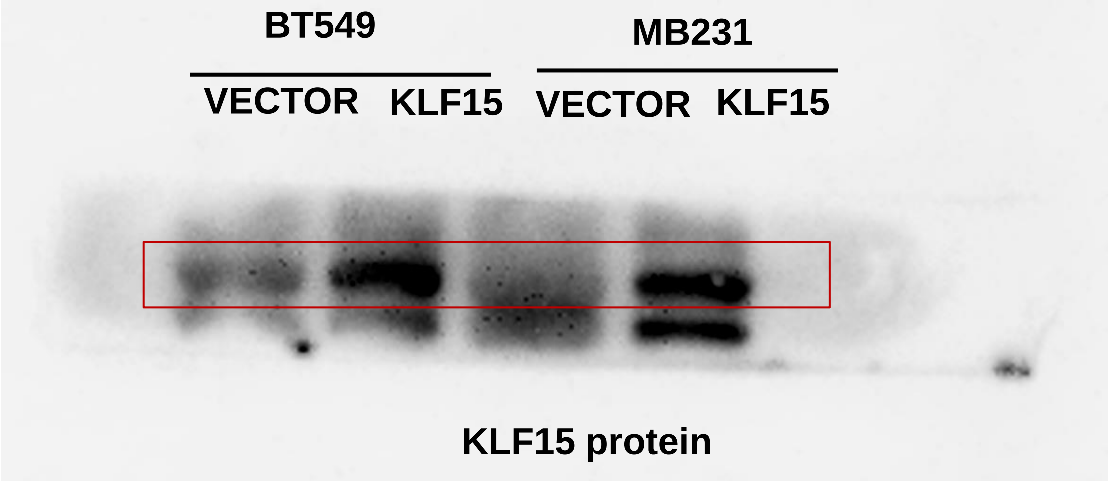

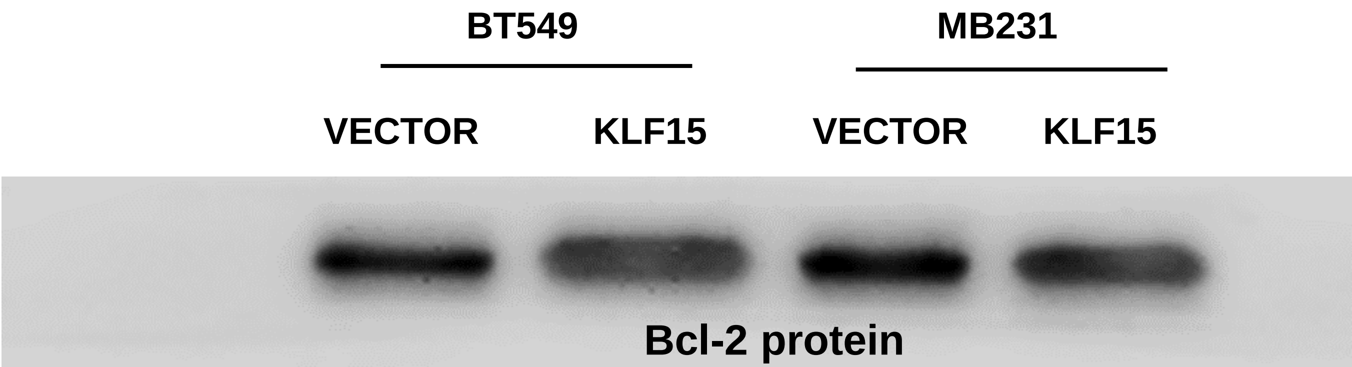

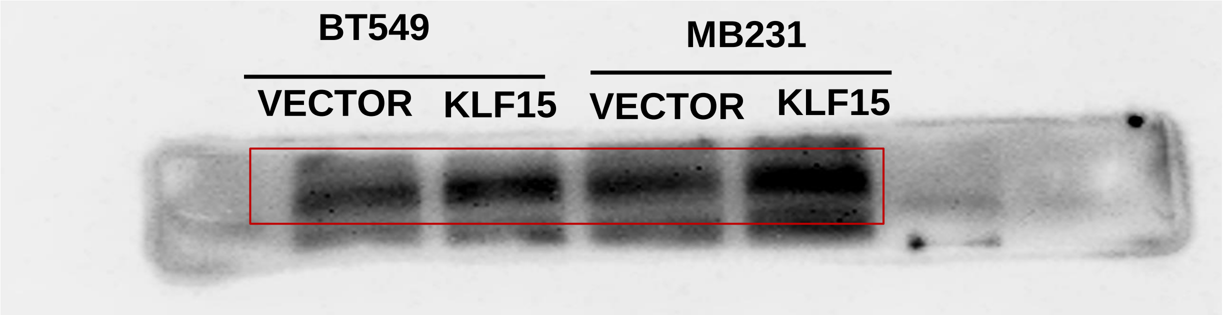

Bax protein

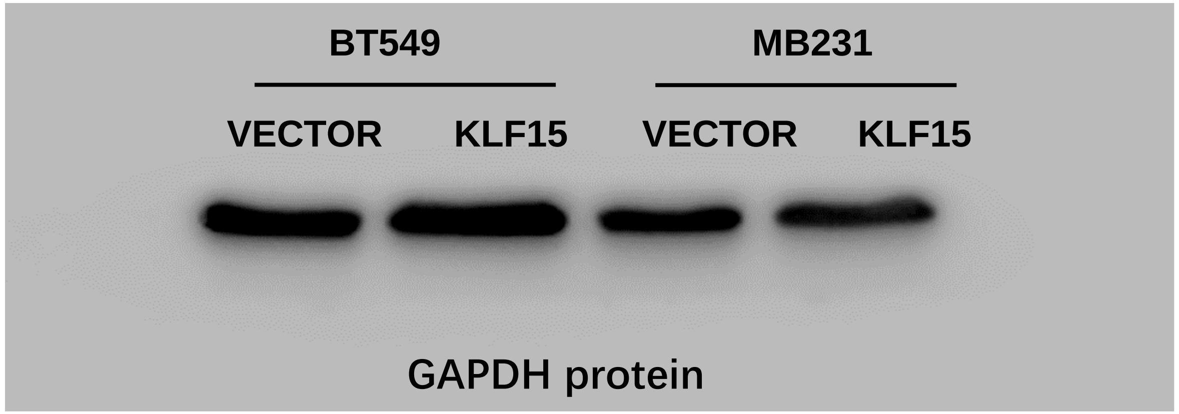

Original Western blots images-Fig 7C

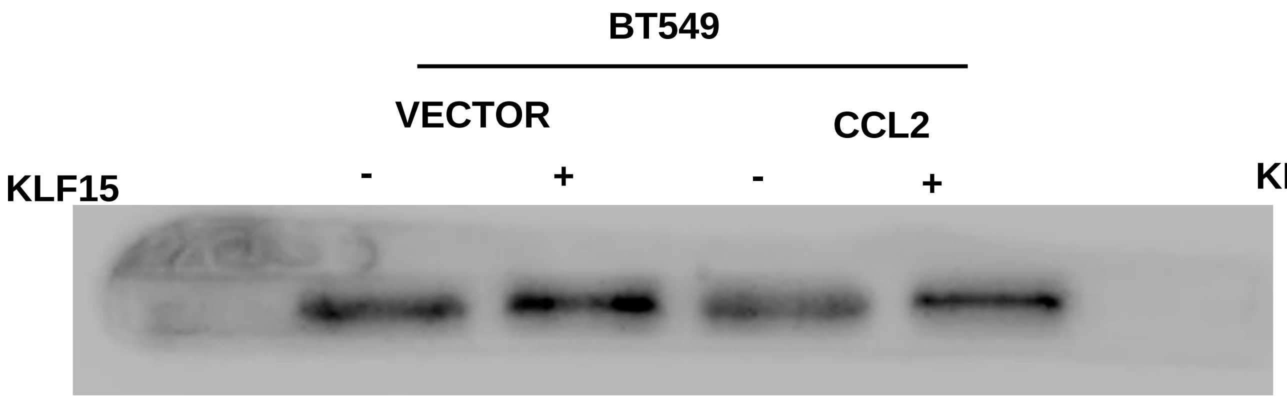

E-cadherin protein

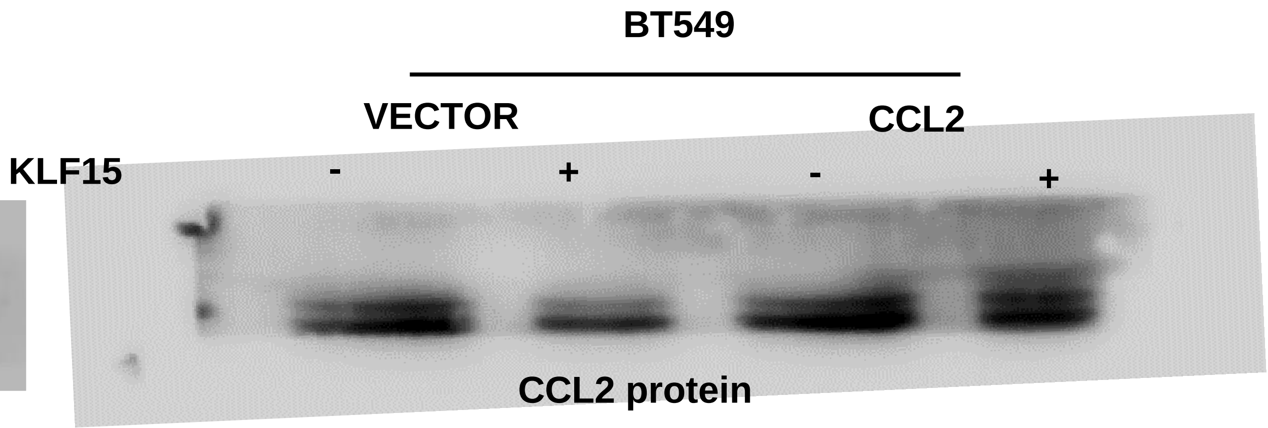

CCL2 protein

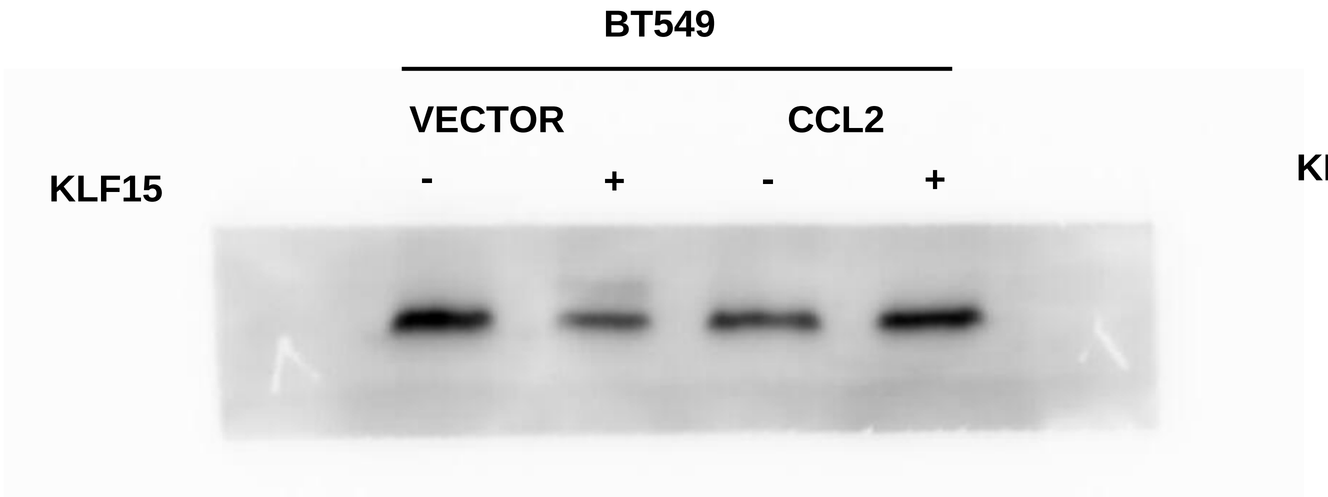

Vimentin protein

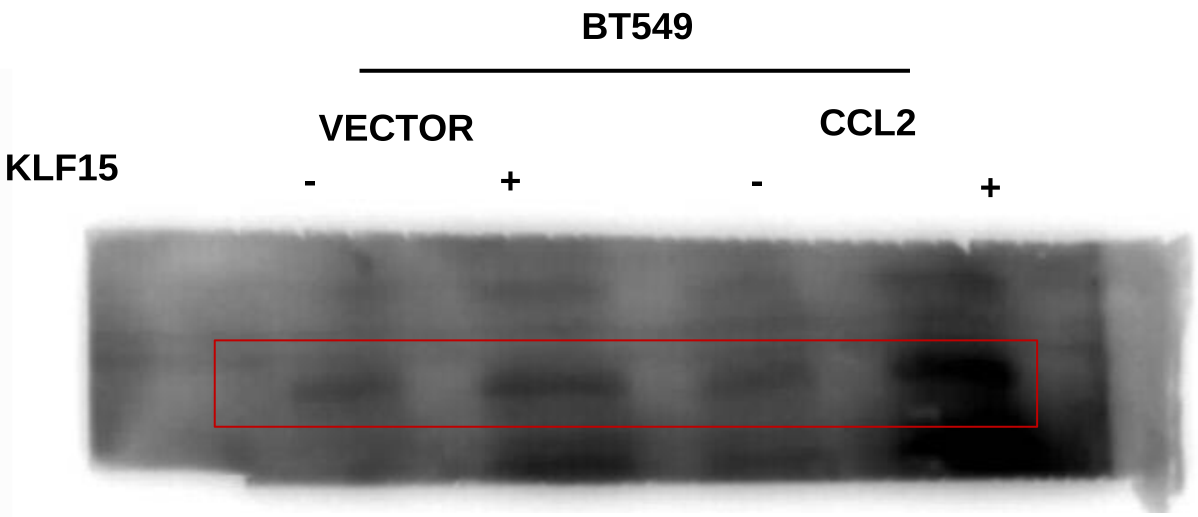

KLF15 protein

Original Western blots images-Fig 7C

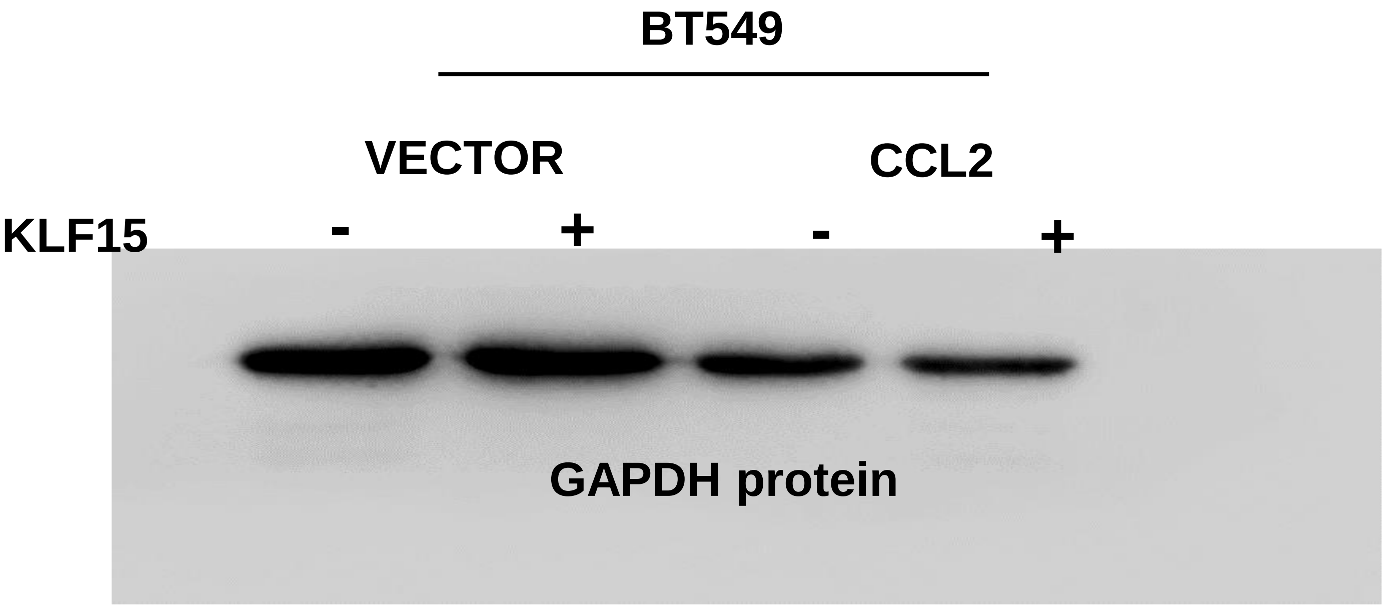

Original Western blots images-Fig 7C

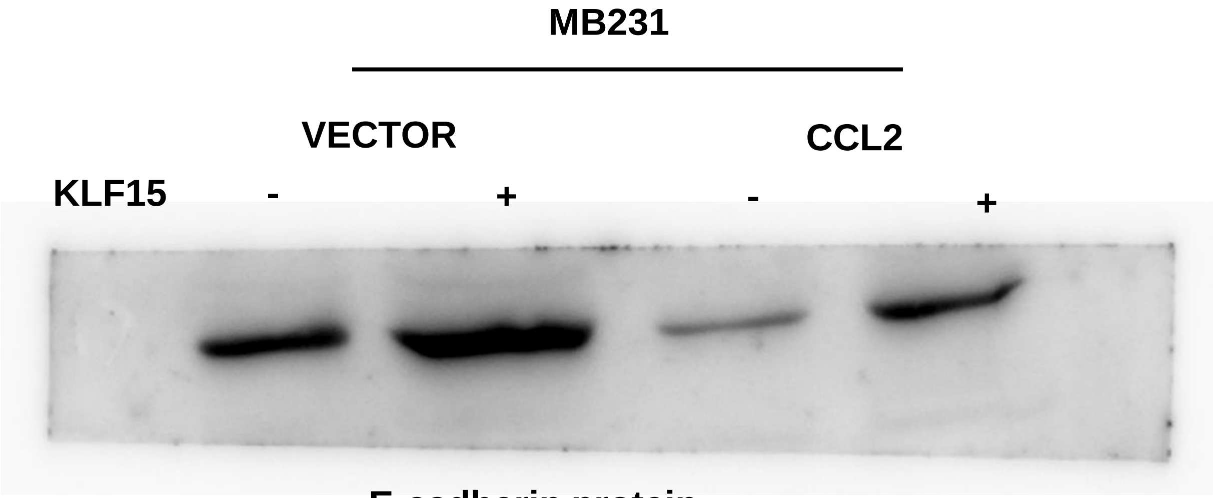

E-cadherin protein

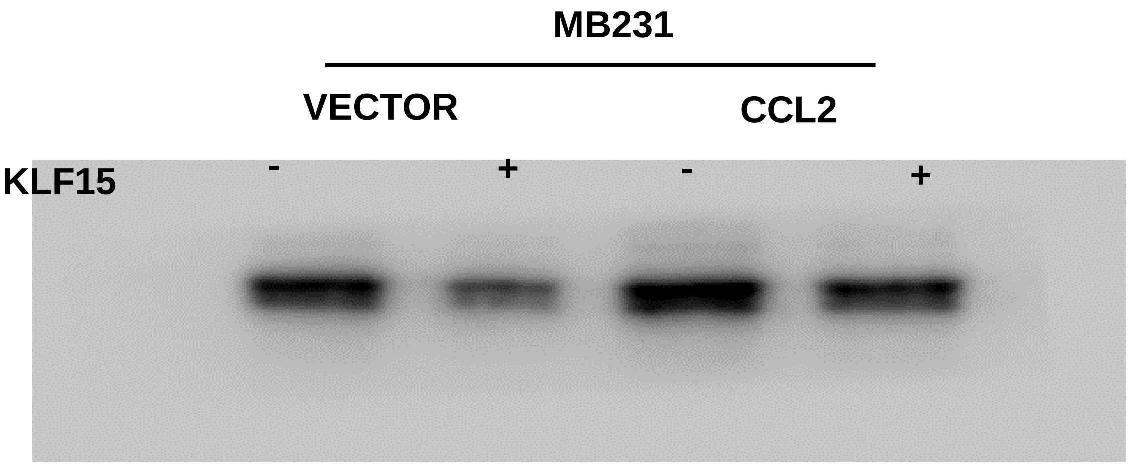

CCL2 protein

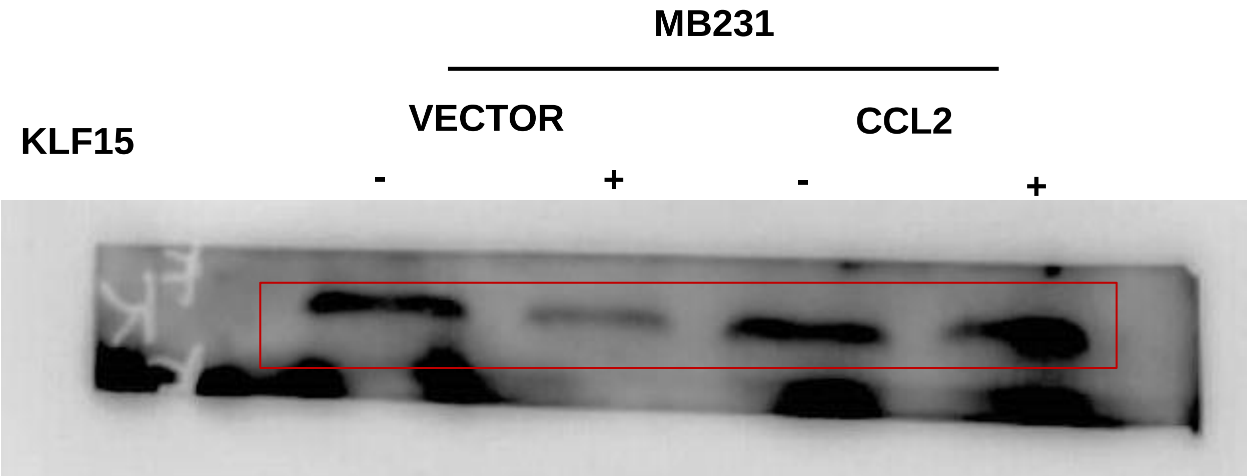

Vimentin protein

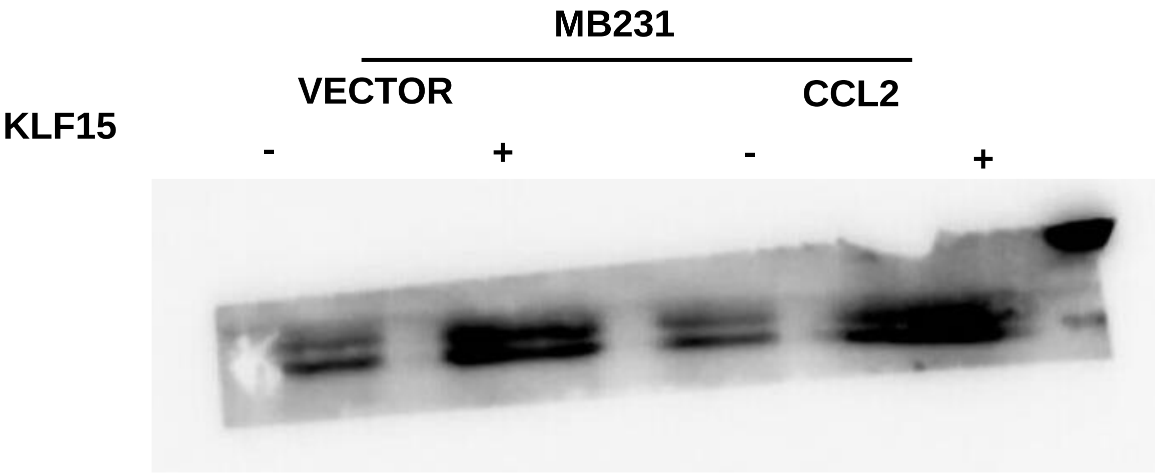

KLF15 protein

Original Western blots images-Fig 7C

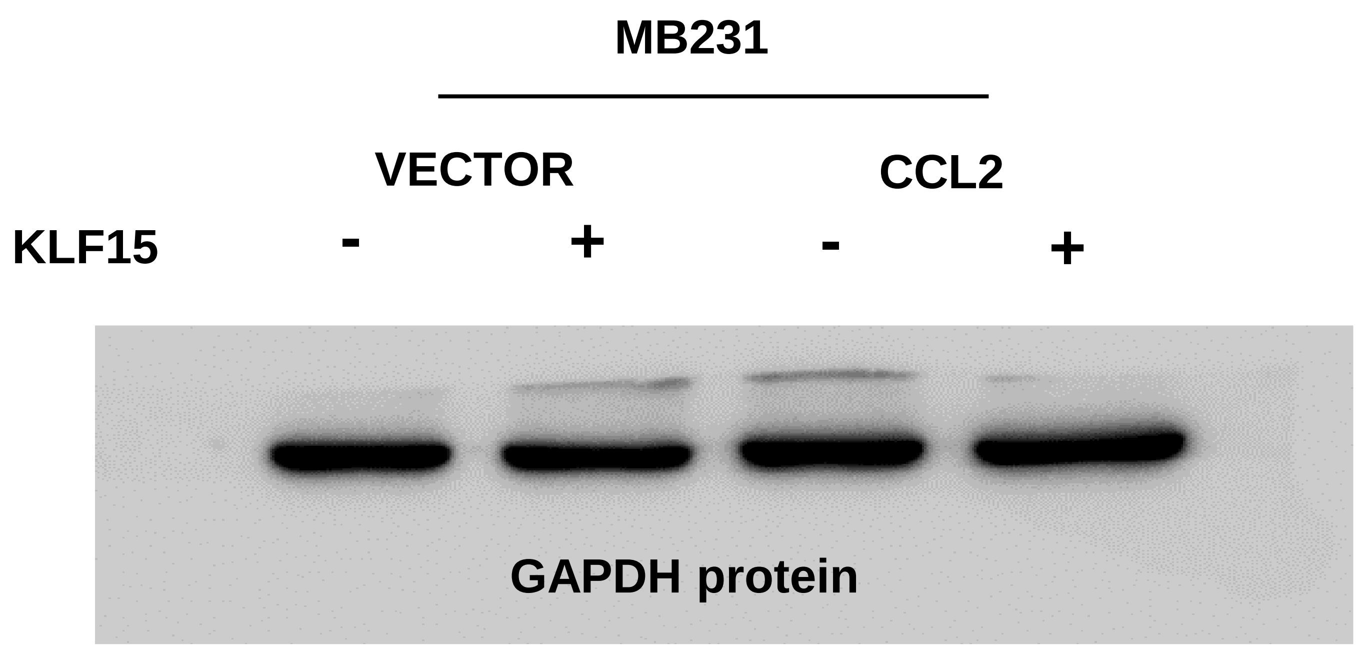

Original Western blots images-Fig 7D

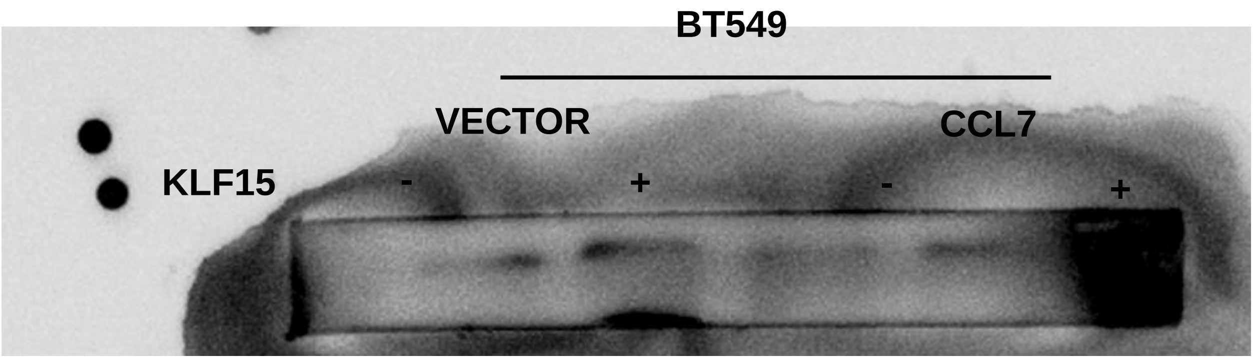

E-cadherin protein

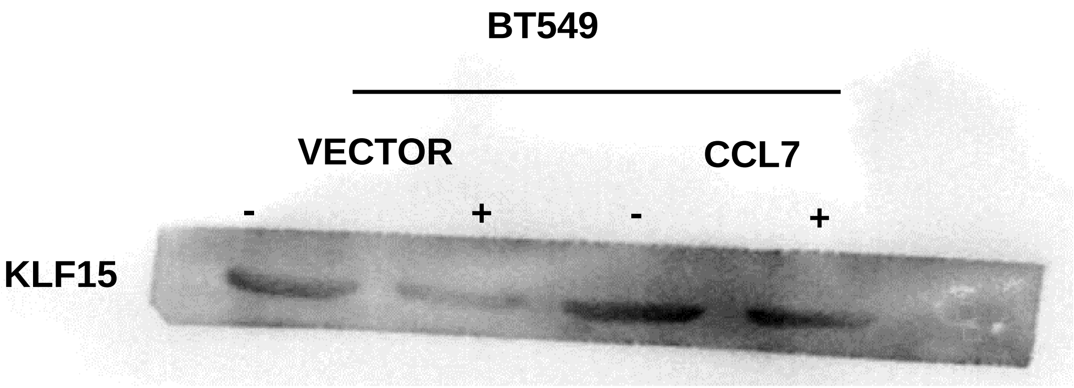

CCL7 protein

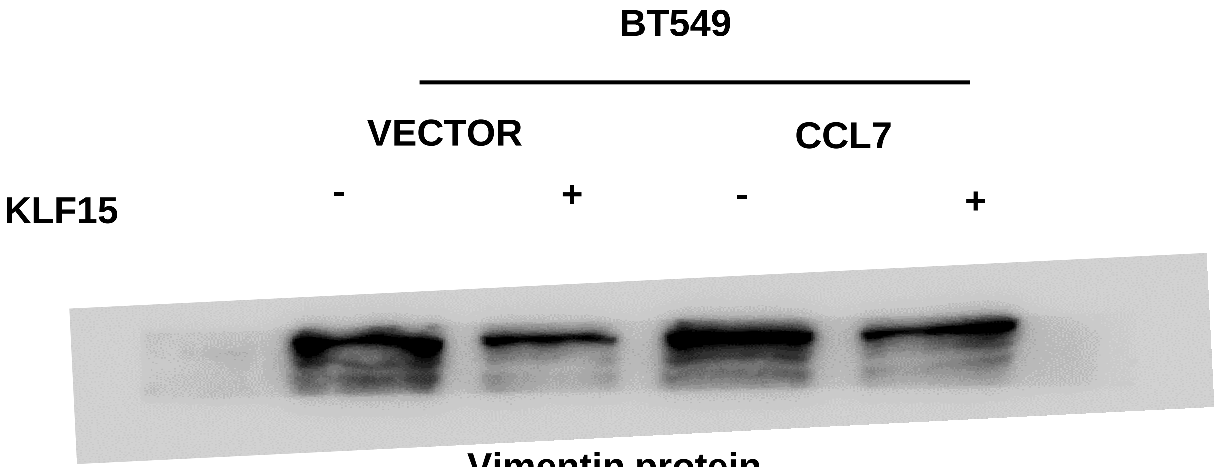

Vimentin protein

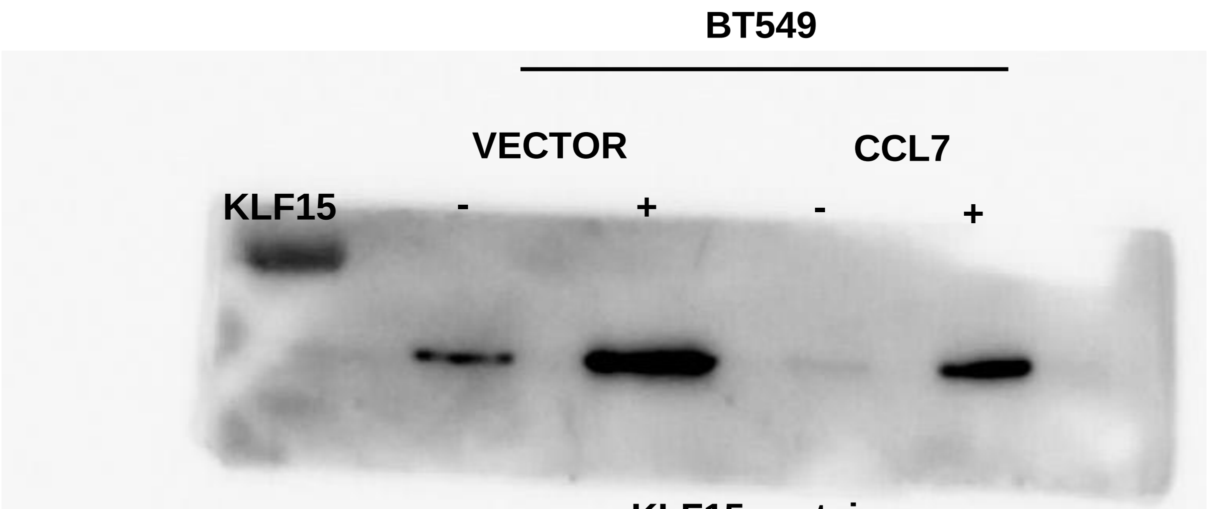

KLF15 protein

Original Western blots images-Fig 7D

BT549

VECTOR CCL7

KLF15 - + - +

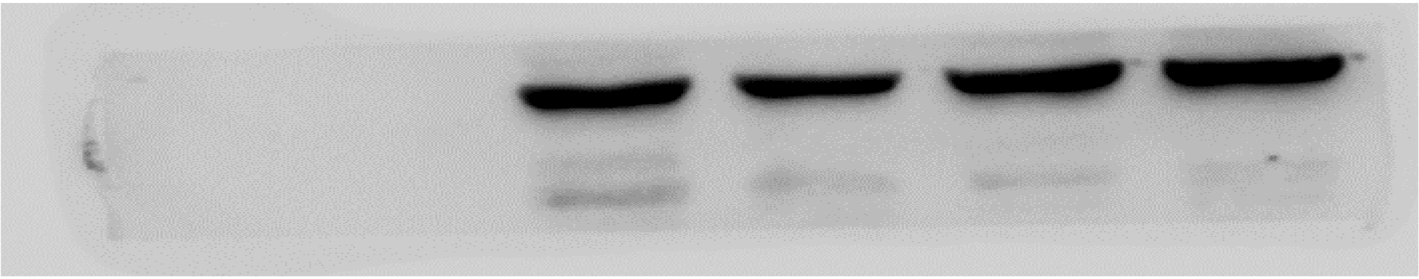

GAPDH protein

Original Western blots images-Fig 7D

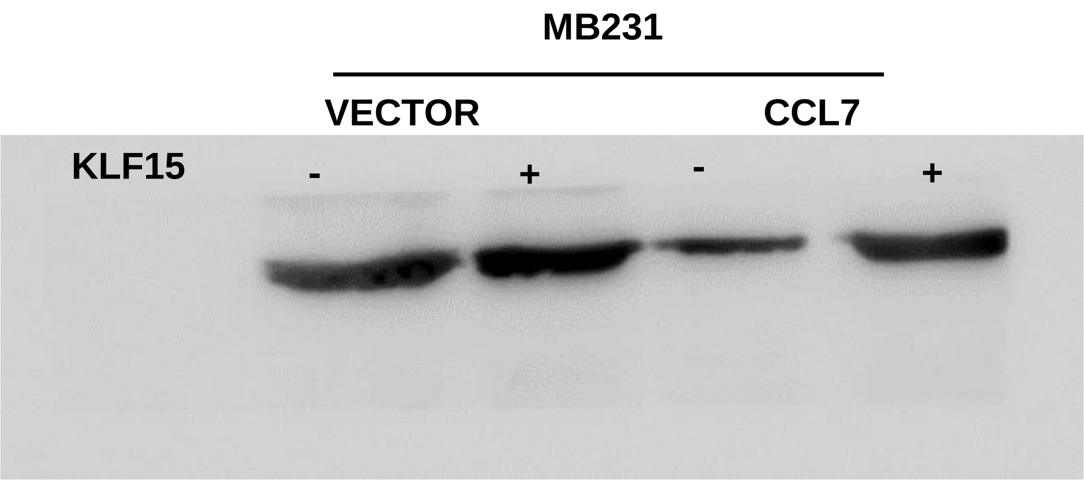

E-cadherin protein

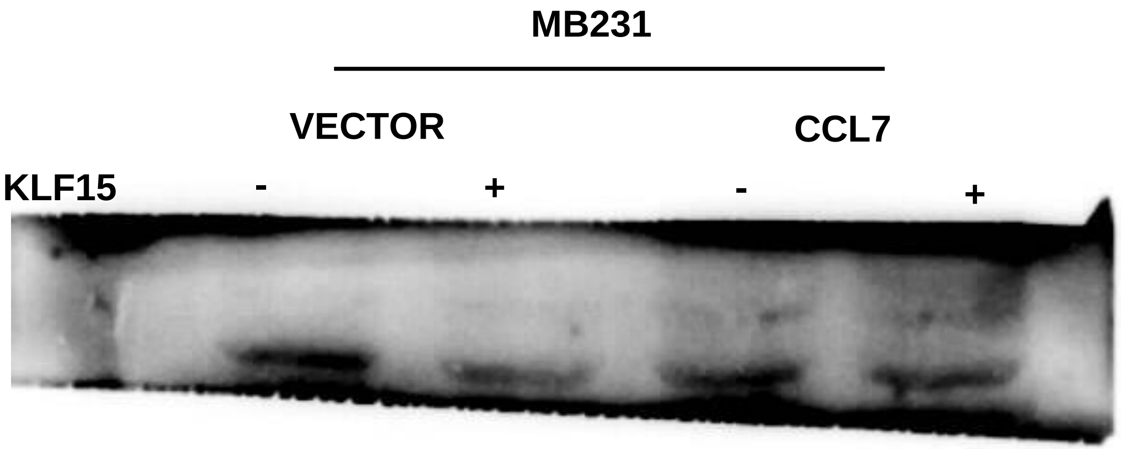

CCL7 protein

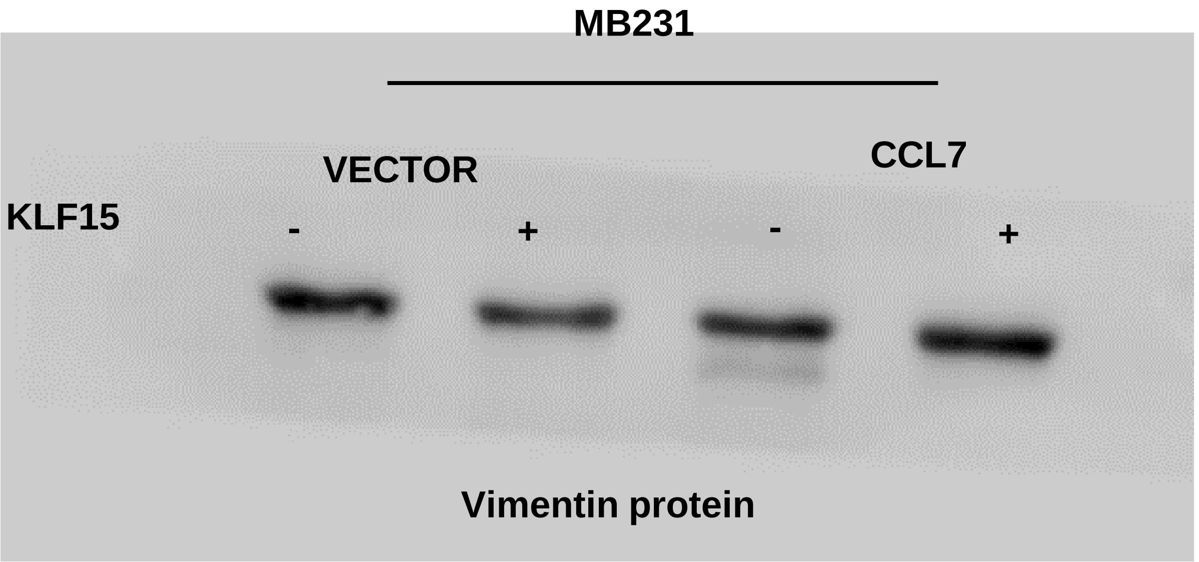

Vimentin protein

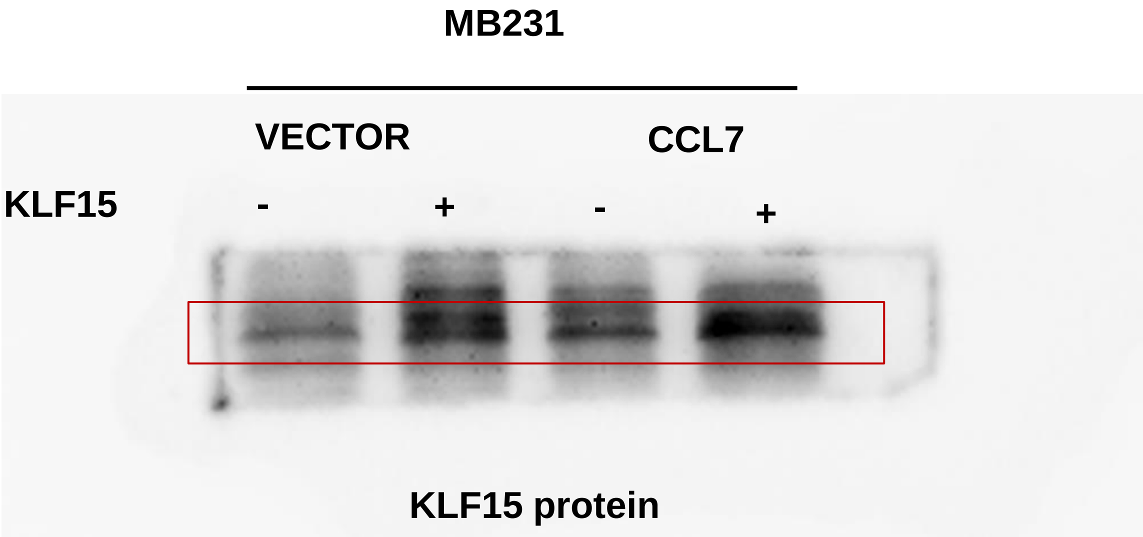

KLF15 protein

Original Western blots images-Fig 7D

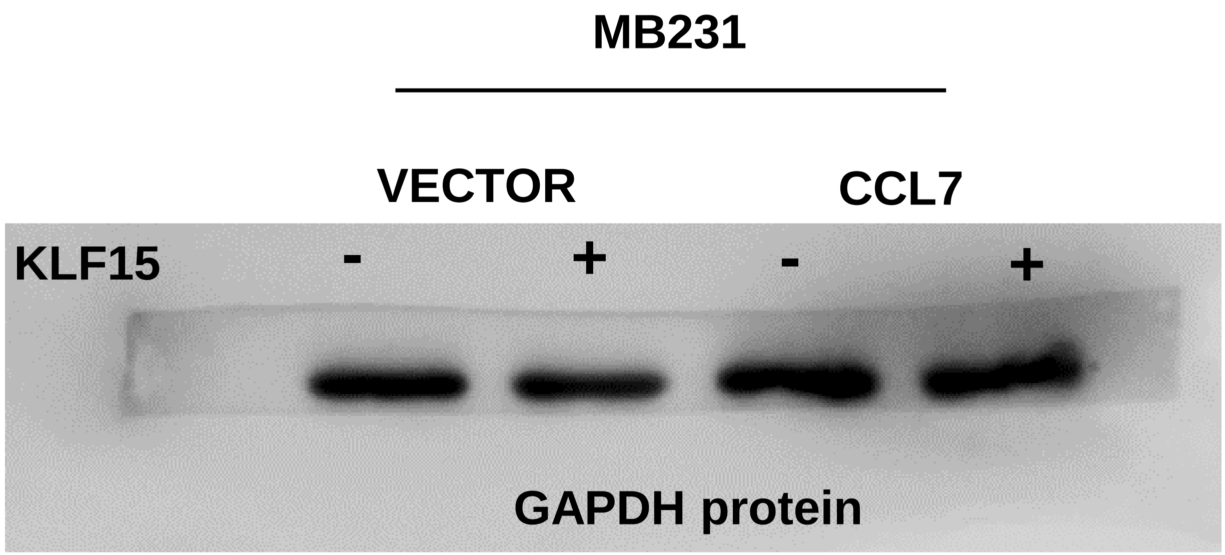

Supplementary Table1. *KLF15* methylation and clinicopathological features of breast tumors

Clinicopathological features	n=192	methylated	unmethylated	p value
**Age**				
<55	108	15	93	0.732
≥55	82	10	72	
Unknown	2		2	
**Tumor size**				
≤5.0 cm	162	22	140	0.713
>5.0 cm	15	2	13	
Unknown	15	1	14	
**Stage (AJCC)**				
I-II	136	19	117	0.798
III-IV	48	6	42	
Unknown	8	0	8	
**Lymph node metastasis**				
Positive	89	12	77	0.953
Negative	91	12	79	
Unknown	12	1	11	
**Distant metastasis**				
Positive	2	0	2	0.578
Negative	170	21	149	
Unknown	20	4	16	
**ER**				
Positive	102	8	94	0.125
Negative	65	10	55	
Unknown	25	7	18	
**PR**				
Positive	81	7	74	0.345
Negative	83	11	72	
Unknown	28	7	21	
**HER2**				
Positive	132	16	116	0.523
Negative	32	2	30	
Unknown	28	7	21	
**P53**				
Positive	75	12	63	0.133
Negative	65	5	60	
Unknown	52	8	44	
**Ki-67**				
Positive	119	13	106	0.780
Negative	15	2	13	
Unknown	58	10	48	

Supplementary Table 2. List of Primers used in the present study

PCR Primer	Sequence (5’-3’)	Product size (bp)	Annealing temperature(℃)
KLF15_F	TATCACATGCTGCCCTCACC		
KLF15_R	GAAGTCCAAGATGCTGTCCTG		
CCL2-F	TCATAGCAGCCACCTTCATTC		
CCL2-R	CCTCTGCACTGAGATCTTCCT		
CCL7-F	TACTTCAACTACCTGCTGC		
CCL7-R	GGCTACTGGTGGTCCTTC		
Ecad-F	TACACTGCCCAGGAGCCAGA		
Ecad-R	TGGCACCAGTGTCCGGATTA		
Vimentin-F	GACCAGCTAACCAACGACAA		
Vimentin-R	GTCAACATCCTGTCTGAAAGAT		
GAPDH -F	GGAGTCAACGGATTTGGT		
GAPDH -R	GTGATGGGATTTCCATTGAT		
CCL2chip-F1	ACAGGATGCTGCATTTGCTCA		
CCL2chip-R1	CTCTGGCTGCTGTCTCTGC		
CCL2chip-F2	GCAGAGACAGCAGCCAGAG		
CCL2chip-R2	GCGAGAGTGCGAGCTTCAG		
CCL7chip-F	CAACTACCTGCTGCTACAGAT		
CCL7chip-R	GTGGTCATCGTCCACATACAT		
KLF15-m1	TGTTTAGCGAGTTGCGGGC	124bp	60
KLF15-m2	AACGACTAAACTCTCGATCCG		
KLF15-u1	AGTGTTTAGTGAGTTGTGGGT	128bp	58
KLF15-u2	ACAACAACTAAACTCTCAATCCA		

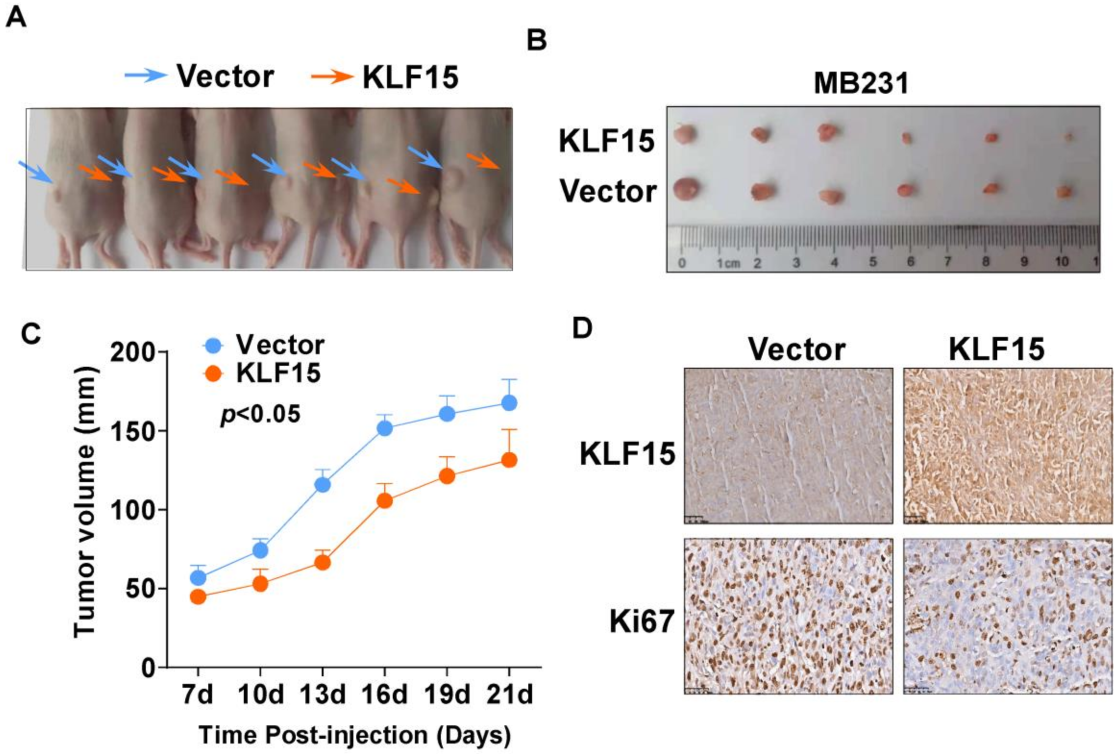

Supplementary Figure 1. **KLF15 inhibits TNBC cells in xenograft nude mice model.** A. Representative imagines of xenograft tumor model on nude mice before resection. Blue arrow: tumors derived from Vector MDA-MB231 cells; Red arrow: tumors derived from KLF15 MDA-MB231 cells. B. Tumors detached from nude mice. C. Tumor growth curve within the 21 days post injection. D. IHC results demonstrating overexpression of KLF15 and decreased Ki67 levels in KLF15 derived tumor tissues.
